# Slow cooling and efficient extraction of C-exciton hot carriers in MoS_2_ monolayer

**DOI:** 10.1038/ncomms13906

**Published:** 2017-01-05

**Authors:** Lei Wang, Zhuo Wang, Hai-Yu Wang, Gustavo Grinblat, Yu-Li Huang, Dan Wang, Xiao-Hui Ye, Xian-Bin Li, Qiaoliang Bao, AndrewThye-Shen Wee, Stefan A Maier, Qi-Dai Chen, Min-Lin Zhong, Cheng-Wei Qiu, Hong-Bo Sun

**Affiliations:** 1 State Key Laboratory on Integrated Optoelectronics, College of Electronic Science and Engineering, Jilin University, 2699 Qianjin Street, Changchun 130012, China; 2 Department of Electrical & Computer Engineering, National University of Singapore, 4 Engineering Drive 3, Singapore 117583, Singapore; 3 Department of Physics, National University of Singapore, 2 Science Drive 3, Singapore 117542, Singapore; 4 The Blackett Laboratory, Department of Physics, Imperial College London, London SW7 2AZ, UK; 5 Institute of Materials Research & Engineering (IMRE), A*STAR (Agency for Science, Technology and Research), 2 Fusionopolis Way, Innovis 138634, Singapore; 6 Laser Materials Processing Research Center, School of Materials Science and Engineering, Tsinghua University, Beijing 100084, China; 7 Department of Materials Science and Engineering, Monash University, Clayton, Victoria 3800, Australia; 8 College of Physics, Jilin University, 2699 Qianjin Street, Changchun 130012, China

## Abstract

In emerging optoelectronic applications, such as water photolysis, exciton fission and novel photovoltaics involving low-dimensional nanomaterials, hot-carrier relaxation and extraction mechanisms play an indispensable and intriguing role in their photo-electron conversion processes. Two-dimensional transition metal dichalcogenides have attracted much attention in above fields recently; however, insight into the relaxation mechanism of hot electron-hole pairs in the band nesting region denoted as C-excitons, remains elusive. Using MoS_2_ monolayers as a model two-dimensional transition metal dichalcogenide system, here we report a slower hot-carrier cooling for C-excitons, in comparison with band-edge excitons. We deduce that this effect arises from the favourable band alignment and transient excited-state Coulomb environment, rather than solely on quantum confinement in two-dimension systems. We identify the screening-sensitive bandgap renormalization for MoS_2_ monolayer/graphene heterostructures, and confirm the initial hot-carrier extraction for the C-exciton state with an unprecedented efficiency of 80%, accompanied by a twofold reduction in the exciton binding energy.

## Abstract (web summary)

Light-matter interaction in atomically thin transition metal dichalcogenides is dominated by excitonic effects and hot-carrier relaxation/extraction mechanisms. Here, the authors report that the C exciton in two-dimensional MoS_2_ exhibits a slower hot-carrier cooling than band-edge excitons.

Control of the relaxation behaviour of electron-hole pairs at the least possible cost is highly desirable for hot carrier-related optoelectronic applications^1^,^2^. In the field of bulk semiconductor photovoltaics, phonon-assisted hot-carrier cooling generates heat, so the maximum theoretical light-into-electricity conversion efficiency is limited to only ∼31% (ref. 3), a value that could double if all hot carriers could be extracted efficiently by suitable conductors^4^. To overcome this drawback, slowing down the hot-carrier relaxation via circumventing the phonon-assisted relaxation is the typical approach investigated^5^.

Reducing the material dimensionality to achieve quantum confinement from one (two-dimensional material) to all three (quantum dot) dimensions is believed to slow hot-carrier cooling, since the enlarged energy spacing arising from quantum confinement causes a ‘phonon bottleneck'^6^. Nevertheless, the initial hot-carrier extraction efficiency in typical quasi-zero-dimensional semiconductor quantum dot systems at room temperature is still low. For CdSe/CdS/ZnS core-shell quantum dots, only about a third of the photogenerated hot electrons located in the CdS shell will transfer to the electron acceptor (methyl viologen)^7^. Moreover, for bare CdSe quantum dots adsorbed within methyl viologen this hot electron extraction is negligible, because an extremely fast hot-carrier extraction process is required to compete against phonon-assisted relaxation^8^. Additional relaxation mechanisms that come into play are Auger-like processes and surface-state trapping, which are efficient in quantum dots^9^,^10^, unless the photogenerated electrons and holes are spatially separated to a certain extent^5^,^7^.

Recently studied two-dimensional systems such as semiconducting transition metal dichalcogenides (TMDCs) with finite bandgaps^11^,^12^ have been shown to be very promising for ultrathin and flexible energy conversion and storage devices^13^,^14^,^15^,^16^,^17^. Due to the parallel bands in their density of states or the so-called band nesting effect^18^, these atomically thin materials present strong optical responses even for excitation energies far exceeding their bandgaps. Recent work in atomically thin molybdenum disulfide (MoS_2_) have suggested that the photoexcited electron-hole pairs in the ‘band nesting' region (denoted as C-excitons) could exhibit a very fast intraband relaxation and a very slow indirect emission process due to spontaneous charge-separation in momentum space^19^,^20^. Hence, it can be expected that the resulting hot-carrier relaxation for the C-exciton state will be different from that associated with the band-edge excitons in atomically thin TMDCs. Moreover, because of the renormalized bandgap and enhanced electron-electron interactions induced by the reduced Coulomb screening^21^, as well as an indirect to direct bandgap transition attributed to the quantum confinement effect^22^,^23^ in two dimensions, this C-exciton relaxation is expected to be distinguishable from its few-layer and bulk counterparts. Indeed, previous time-resolved studies have shown that the intervalley (K−Γ) transfer in MoS_2_ bulk is very efficient due to the dominant indirect bandgap characteristic^24^, while for the few-layer MoS_2_ case, although there is efficient charge carrier photogeneration^25^, the decay of neutral C-excitons is still faster than neutral band-edge excitons. However, the fundamental photophysical mechanism and detailed processes of hot-carrier relaxation and follow-up extraction for the C-exciton state in single-layer TDMCs have not yet been elucidated^26^, and could be crucial to understand the work mechanisms in related two-dimensional nanophotonic devices.

In this article, we report the transient characteristics of the intrinsic high-energy C-exciton state in MoS_2_ monolayer and MoS_2_ monolayer/graphene heterostructure (Fig. 1a) by studying its femtosecond broadband transient absorption (TA) spectrum. We observe a slower hot-carrier relaxation for the C-excitons, in comparison with band-edge A*/*B-excitons, indicating the presence of an additional mechanism for the suppression of phonon-assisted relaxation which is not dominated by the quantum confinement effect. In combination with scanning tunnelling spectroscopic (STS) analysis and steady-state optical spectroscopies, we identify the screening-sensitive bandgap renormalization of single-layer MoS_2_ with and without graphene as a substrate. The relevant initial charge transfer processes in MoS_2_ monolayer/graphene heterostructures are confirmed, and a direct hot-carrier extraction with a remarkable efficiency of ∼80% is demonstrated for the C-exciton at room temperature, with corresponding values for A/B-excitons of 93%/81%. These results show that atomically thin TMDCs provide opportunities for modifying the electronic structure and manipulating the hot-carrier relaxation behaviour in two-dimensional *d*-electron systems.

## Results

### Steady-state optical characterization

MoS_2_ monolayers and graphene were grown by chemical vapour deposition (CVD) on SiO_2_/Si substrates and copper foils, respectively^27^, and then transferred to a fresh quartz substrate using a standard poly(methylmethacrylate) (PMMA) transfer method^28^. Details of MoS_2_ monolayer and MoS_2_ monolayer/graphene sample preparation and steady-state characterizations are presented in the Methods section. To confirm the sample quality, optical microscopy and atomic force microscopy studies, as well as steady-state photoluminescence (PL) and Raman characterization, were performed, as shown in Supplementary Figs 1−4.

**Figure 1:**
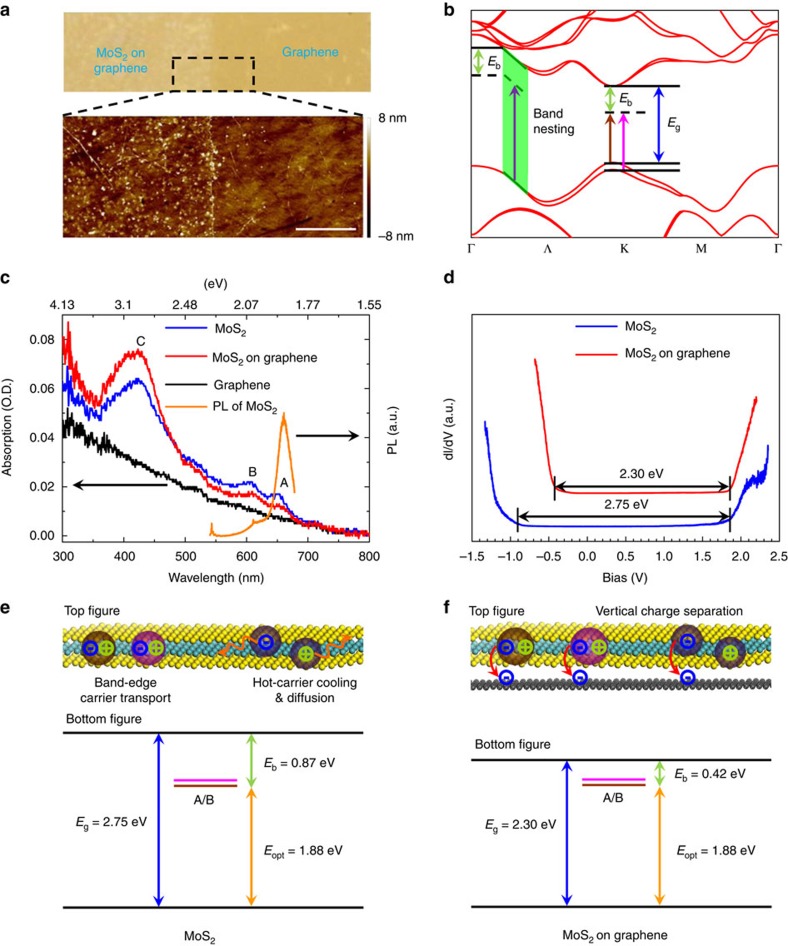
Influence of graphene on the electronic bandgap of MoS_2_ monolayer. (**a**) Optical microscope and AFM images of MoS_2_ monolayer/graphene heterostructure. In the bottom, the scale bar is 2 μm. (**b**) Theoretically predicted energy band structure corresponding to the direct transitions in the momentum space of MoS_2_ monolayers considering the spin-orbit coupling. *E*_g_, energy gap; *E*_b_, exciton binding energy. The green area is the band nesting region. The vertical brown, pink and purple arrows in **b** represent the optical transitions of A/B/C-exciton, respectively. (**c**) Steady-state absorption spectra of graphene, MoS_2_ monolayer and MoS_2_ monolayer/graphene heterostructure. O.D., optical density. The orange solid line is the PL spectrum of MoS_2_ monolayer under 532 nm excitation. (**d**) STS spectra for MoS_2_ monolayer and MoS_2_ monolayer/graphene heterostructure. (**e**) Sketch of electronic and optical bandgaps for MoS_2_ monolayer (bottom figure). The top image illustrates the MoS_2_ monolayer (sky blue solid sphere represents the Mo atom, and yellow solid sphere represents the S atom); brown, pink and purple spheres represent the A/B/C-exciton, respectively; the orange curves represent the hot-carrier cooling and diffusion processes. (**f**) Sketch of electronic and optical bandgaps for MoS_2_ monolayer/graphene heterostructure (bottom figure). The top illustration schematizes the MoS_2_ monolayer/graphene heterostructure (black solid sphere represents the C atom); the red arrows indicate the vertical electron extraction processes. *E*_opt_, optical bandgap.

Figure 1b,c exhibit, respectively, the *ab initio* calculation of the band structure related to the band nesting region for MoS_2_ and the corresponding measured steady-state absorption and PL spectra. According to Fig. 1c, when MoS_2_ monolayers are placed on a graphene substrate, the oscillator strengths of band-edge A/B-excitons around 650 and 600 nm decrease slightly, while for the high-energy C-exciton around 430 nm the oscillator strength increases. The resulting PL of the MoS_2_ monolayer/graphene heterostructure (not shown) is found to be strongly quenched, in contrast to the reference case of MoS_2_ monolayers alone (Fig. 1c), where the PL peak at around 660 nm (1.88 eV) is observed, corresponding to the optical bandgap (*E*_opt_) of the MoS_2_ monolayer. These results imply complex interactions between the MoS_2_ monolayer and substrates^29^ that need to be understood.

### Electronic bandgap measurement and transient experiment

To shed light on the correlations between the electronic structures of MoS_2_ monolayer on different substrates and internal exciton relaxations, electronic bandgap and time-resolved ultrafast spectroscopy studies were performed. The influence of graphene on the electronic bandgap (*E*_g_, equal to the quasiparticle bandgap, *E*_q_) of MoS_2_ monolayer is revealed by the STS analysis shown in Fig. 1d. It is found that *E*_g_ is reduced from 2.75 eV for MoS_2_ monolayer alone to 2.30 eV for MoS_2_ monolayer/graphene heterostructure. Moreover, the measurements indicate that graphene lowers the conduction band minimum (CBM, left) of the MoS_2_ monolayer by ∼0.45 eV, while the corresponding valence band maximum (VBM, right) is pinned at ∼1.85 eV below the Fermi level. The exciton binding energy, *E*_b_=*E*_g_–*E*_opt_, is calculated to be 0.42 and 0.87 eV for the MoS_2_ monolayer with and without graphene, respectively. The former value approximates well to the previously reported values of *E*_b_ of 0.48 eV and associated *E*_g_ of 2.40 eV for CVD MoS_2_ monolayers grown directly on cleaved graphite (HOPG) substrates^30^, and the latter is close to the reported theoretical predictions (*E*_b_ of 1.0 eV and *E*_g_ of 2.8 eV) (refs 31, 32, 33, 34, 35). This indicates a screening-sensitive bandgap renormalization, similar to that reported for the MoSe_2_ monolayer^21^. In addition, the reduced *E*_b_ measured for MoS_2_ monolayer/graphene heterostructures could facilitate the charge transfer from MoS_2_ monolayer to graphene, as will be discussed later. The relevant energy levels for MoS_2_ monolayer and MoS_2_ monolayer/graphene heterostructure are presented in Fig. 1e,f, respectively, as extracted from the steady-state experimental data. According to our experimental observations and density functional theory (DFT) calculations (Supplementary Fig. 5), the location of Dirac point of graphene in energy is close to the middle of bandgap of MoS_2_ monolayer, about 1.20 eV higher than its VBM.

**Figure 2:**
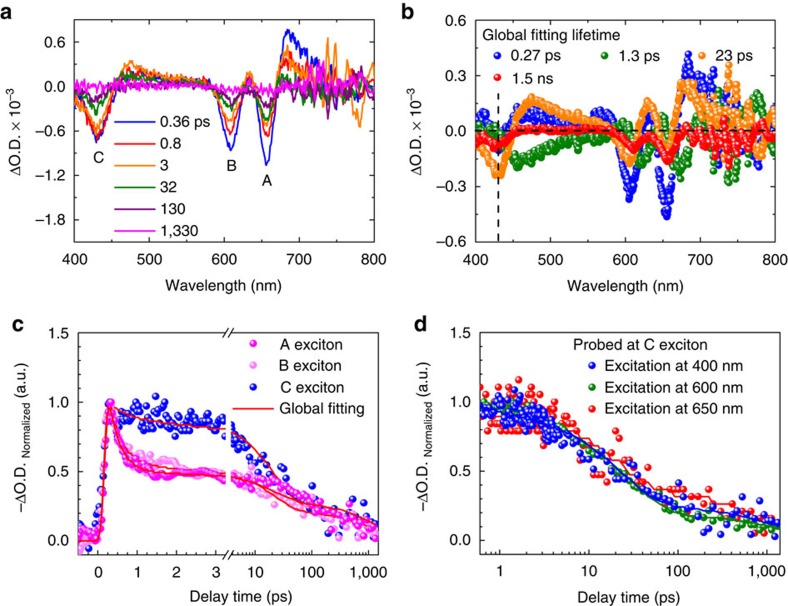
Global fitting for transient absorption spectra of MoS_2_ monolayers. (**a**) Transient absorption spectra of MoS_2_ monolayers probed at different delay times under 400 nm excitation (pump density of 5 μJ cm^−2^, initial exciton density of 1.28 × 10^12^ cm^−2^). (**b**) Global analysis for the transient absorption data in **a**. The vertical dashed line indicates the position of the C-exciton state, while the horizontal dashed line indicates ΔO.D.=0. (**c**) Normalized decay dynamics of A-, B- and C-exciton states under the pump condition in **a**. The red solid lines are the global fitting results. (**d**) Normalized decay dynamics of the C-exciton state under 400, 600 and 650 nm excitation with similar initial exciton density. The solid lines are the global fitting results.

The inherent excited-state relaxation processes for A/B/C-excitons in MoS_2_ monolayers and MoS_2_ monolayer/graphene heterostructures are elucidated by broadband TA experiments in transmission mode^36^,^37^. Typical TA spectra of MoS_2_ monolayers under 400 nm excitation (pump density of 5 μJ cm^−2^, initial exciton density of 1.28 × 10^12^ cm^−2^) are shown in Fig. 2a. These spectra represent the pump-induced absorption changes observed in the samples, namely the differential optical density with and without pump light excitation (ΔO.D.=O.D._pump_—O.D._w/o pump_). As it can be seen in Fig. 2a, there are three distinct negative transient species (bleaching signals) at around 656, 608 and 430 nm (1.89, 2.04 and 2.88 eV), corresponding to the energy states of A/B/C-excitons observed in the steady-state absorption spectrum (Fig. 1c), respectively. The positive peaks for atomically thin TMDCs as shown in Fig. 2a are usually attributed to peak shift and broadening of A/B/C-exciton states^38^,^39^,^40^ (Supplementary Fig. 6 and Supplementary Note 1), excited-state absorption and/or the absorption of new photogenerated transient species, for example, the effect of charges at high pump conditions^25^,^26^.

Through a global analysis [ΔO.D.=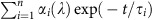, where *α*_*i*_(*λ*) are the pre-exponential factors used for the calculation of decay-associated spectra (DAS), *λ* is probe wavelength and *τ*_*i*_ are *λ*-independent lifetimes] (refs 37, 41), as shown in Fig. 2b, it is deduced that there are at least four lifetime components for MoS_2_ monolayers. For the band-edge bleaching region, the first two shortest lifetime components mainly represent the contributions of A/B-exciton populations. The lifetime component of 0.27 ps (blue) that is dominant in this spectral region could arise from fast nonradiative recombination, for example, exciton-exciton annihilation^42^,^43^, while the next lifetime component of 1.3 ps (green), since not only matches the spectral positions of steady-state PL peaks well, but also agrees with the fast decay component of band-edge emission^44^, it could be responsible for the radiative recombination of band-edge excitons. Concerning the bleaching region of the high-energy C-exciton state, the longer lifetime component of 23 ps (orange) is dominant, and could represent contributions of charge populations generated by self-separation of C-excitons in momentum space forming hot carriers. Finally, the remaining lifetime component in the nanosecond range (red) is used to match the long-lifetime decay background. It is therefore suggested that the global analysis on the TA data of MoS_2_ monolayer should be applied when the initial exciton density is low enough, for example, less than 2 × 10^12^ cm^−2^ adopted here.

This analysis demonstrates a slower decay for the hot-carriers associated with the C-exciton state, especially at the first tens of picoseconds, as observed in Fig. 2c. It is worth noting that this anomalous relaxation behaviour is contrary to the expected faster decay for the C-exciton when considering only its electronic instability. In fact, the parallel bands in the density of states will induce very fast carrier relaxation, as we demonstrate in twisted bilayer graphene (see detailed discussions in Supplementary Figs 7,8 and Supplementary Note 2), where the average carrier lifetime is only ∼0.2 ps. However, for MoS_2_ monolayers, in the case the excited-states of lower energy band-edge exciton states are already occupied, the C-exciton state under 400 nm excitation could cause a transient and complex excited-state Coulomb environment in the pump spectral region. In this context, the high-energy C-exciton relaxation in the band nesting region will slow down.

To verify this hypothesis, we performed band-edge excitation TA experiments using 600/650 nm excitation (Supplementary Fig. 9), for which the high-energy C-exciton state is still observed (that is, there is an efficient up-conversion process). For these band-edge excitation conditions, the initial exciton densities were set close to that for the 400 nm excitation case as presented in Fig. 2a-c. The global fitting results reveal subtle differences between the band-edge excitation and 400 nm excitation TA experiments (see detailed discussions in Supplementary Fig. 9, Supplementary Note 3), suggesting a fast tunnelling mechanism from the high excited states of band-edge excitons to the C-exciton state^35^, which is consistent with our hypothesis (Supplementary Fig. 10 and Supplementary Note 4). The resulting characteristic population decay dynamics for the C-exciton state generated by the different excitations give an average hot-carrier lifetime of 350±50 ps (Fig. 2d).

**Figure 3:**
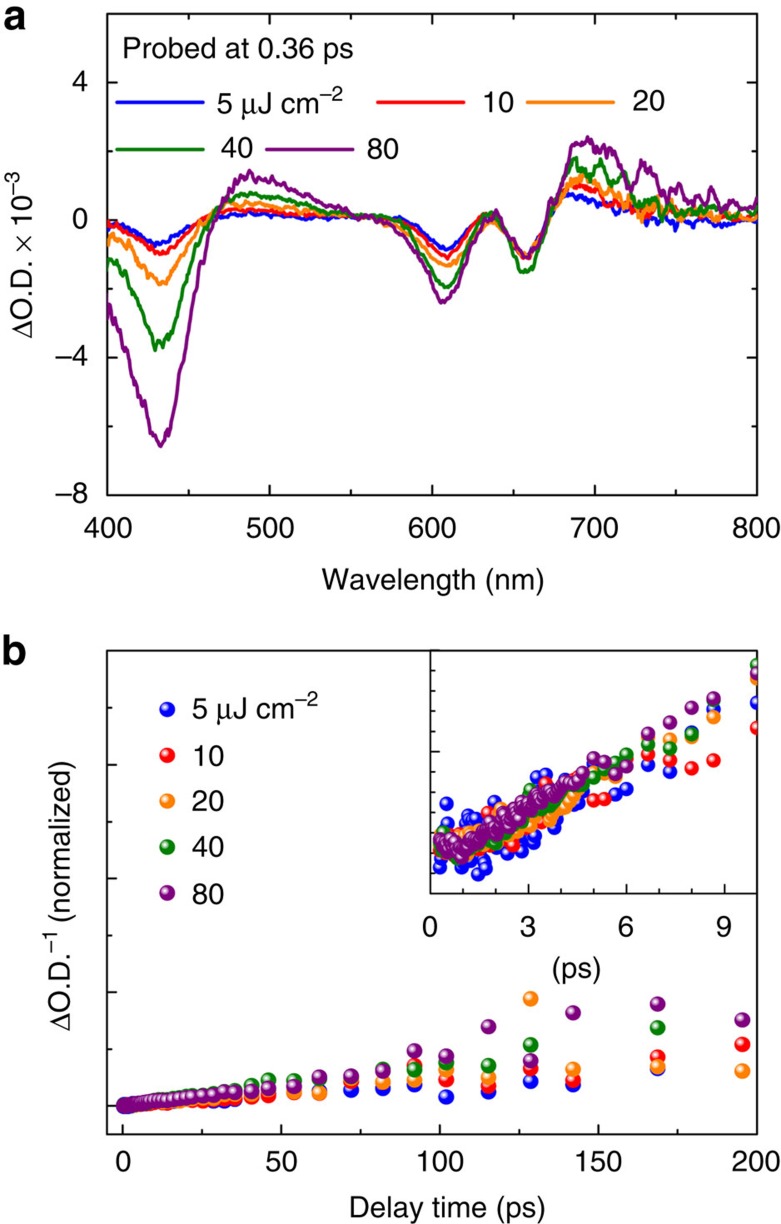
Excitation-density dependent transient behaviours on MoS_2_ monolayers. (**a**) Transient absorption spectra of MoS_2_ monolayers probed at 0.36 ps under 400 nm excitation with different excitation power densities. (**b**) Reciprocal of decay traces of the C-exciton state (430 nm) under different excitation power densities, normalized at the minimum ΔO.D.^−1^. Inset shows the traces in the main panel within the first 10 ps.

TA experiments for higher exciton densities at 400 nm pump condition reveal another remarkable characteristic of the C-exciton state, which is an extremely weak initial exciton-density dependent relaxation. As shown in Fig. 3a, by increasing the pump density from 5 to 80 μJ cm^−2^ (initial exciton density ∼10^12^−10^13^ cm^−2^), the initial TA signal amplitude of the C-exciton state probed at 0.36 ps increases and exhibits a linear behaviour even up to 240 μJ cm^−2^ (Supplementary Fig. 11a). Moreover, while the normalized decay traces at different pump densities for the C-exciton state show similar profiles (Supplementary Fig. 11b), the band-edge bound optical exciton states are strongly affected by the high initial exciton density (see detailed discussions in Supplementary Figs 12,13 and Supplementary Note 5). This implies a transient and reversible method to control the optical responses of band-edge excitons in ultrashort timescales^45^ by changing the carrier population in the C-exciton state.

### Free-carrier relaxation feature for C-exciton hot carriers

Based on the rate equation^46^, describing the non-geminate/free carrier recombination, 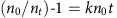, where *n*_0_ is the initial exciton density, *t* is time and *k* is the second-order rate constant for recombination, we find a linear relationship between ΔO.D.^−1^ at the C-exciton state and *t* in Fig. 3b. This linearity is consistent with a two-body recombination mechanism, and supports our hypothesis that photocarriers in the C-exciton state are hot carriers with free-carrier properties. In addition, this linearity is weakly dependent on the initial exciton density, which suggests that the exciton dissociation occurs efficiently in the C-exciton state, in agreement with the self-separation of photocarriers in the nesting region in momentum space. From linear fits using the rate equation at different pump densities, we estimate a second-order recombination rate constant of (3.8±0.2) × 10^−2^ cm^2^ s^−1^ at the extremely low initial exciton density limit (*n*_0_→0) by extrapolating an exponential-decay phenomenological expression, as shown in Supplementary Fig. 11c.

We now turn to the detailed description of hot-carrier relaxation processes in single-layer MoS_2_. It is well known in low-dimensional systems that many-body processes represent an important obstacle for achieving slow hot-carrier cooling. For the anomalous C-exciton relaxation in two-dimensional TMDCs, the band nesting effect is crucial, as it does not only provide an alternative high-energy exciton state, but also promotes self-separation of photocarriers in the nesting region in momentum space, which helps circumvent the Auger recombination channel owing to momentum mismatching. The quantum confinement in the two-dimensional system also changes the energy level alignment between the C-exciton and band-edge optical exciton (A/B-exciton) states. As the conduction and valence bands' energy levels for the former are higher than those for the latter, hot carriers in the C-exciton state will eventually jump to the lowest energy states in momentum space. This process will then dominate over the indirect bandgap recombination that predominates in bulk and few-layers cases. Furthermore, when photoinduced band-edge excitons and their excited states generate C-excitons by an up-conversion/many-body process^26^,^35^, they also play a role in slowing down the C-exciton relaxation due to the transient excited-state Coulomb interactions^47^,^48^. By those factors together, a free-carrier recombination feature is created for the hot carriers in the C-exciton state of MoS_2_ monolayer.

### Initial extraction process for C-exciton hot carriers

**Figure 4:**
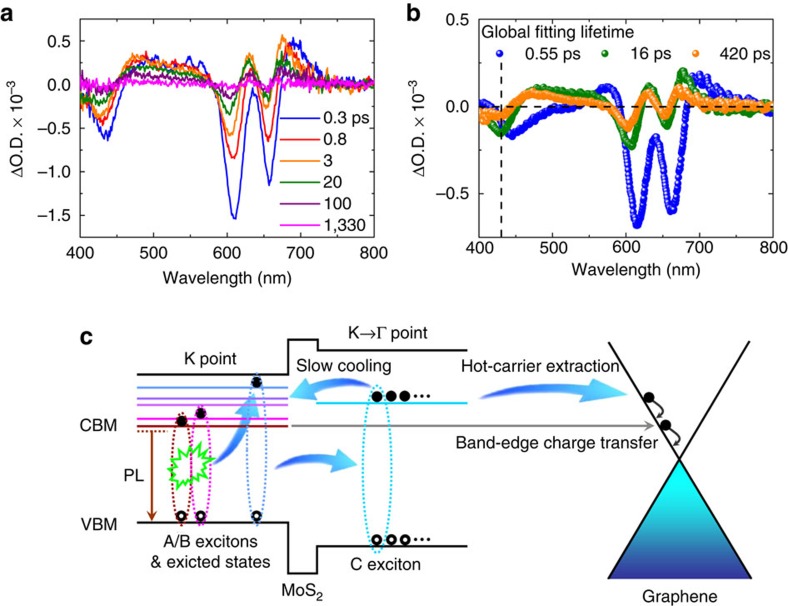
Initial carrier extraction in MoS_2_ monolayer/graphene heterostructure. (**a**) Transient absorption spectra of MoS_2_ monolayer/graphene heterostructures probed at different delay times under 400 nm excitation (initial exciton density of 1.73 × 10^12^ cm^−2^). (**b**) Global analysis for the transient absorption data in **a**. The vertical dashed line indicates the position of the C-exciton state, while the horizontal dashed line indicates ΔO.D.=0. (**c**) Illustration of the photophysical processes in the MoS_2_ monolayer and initial charge transfer in the MoS_2_ monolayer/graphene heterostructure.

Finally, we use the MoS_2_ monolayer/graphene heterostructure to examine the initial carrier extraction processes. Figure 4a,b shows the TA spectra of MoS_2_ monolayer/graphene films at room temperature (400 nm excitation, initial exciton density of 1.73 × 10^12^ cm^−2^) and global fitting results, respectively. It is found that due to the influence of graphene, only three lifetime components compared to four previously are needed in the global analysis. Comparing with the results in bare MoS_2_ monolayers, we calculate that the heterostructures present an initial carrier extraction efficiency of 93%, 81% and 80% for the A-, B- and C-exciton states, respectively. Detailed fitting data are presented in Supplementary Table 1. The normalized dynamics of A-, B- and C-exciton states with and without the graphene substrate are also shown in Supplementary Fig. 14. For the band-edge A-exciton, a high charge transfer efficiency (>90%) is expected, as reported in other atomically thin van der Waals heterojunctions^49^,^50^. In contrast, for the initial hot-carrier extraction in the C-exciton state, this is the first time to our knowledge that such a highly efficient hot-carrier transfer is demonstrated in the MoS_2_ monolayer/graphene heterostructure. The related physical picture is illustrated in Fig. 4c. This observation could open new avenues for hot-carrier-driven optoelectronic applications^2^, such as water photolysis and singlet exciton fission based on atomically thin TMDCs, when other special electron acceptors are utilized to replace the graphene adopted here.

## Discussion

One novel transient feature found here is the slower carrier relaxation (∼350 ps) compared to the band-edge A*/*B-excitons, which stems from the favourable band alignment and complex excited-state Coulomb environment, rather than solely on quantum confinement in two-dimension systems. As a result, the hot-carrier relaxation in the C-exciton state is three orders of magnitude slower than the typical carrier relaxation for parallel bands in the density of states (such as ∼0.2 ps in twisted bilayer graphene). Thus, two lateral carrier transport modes in MoS_2_ monolayers are revealed: slow hot-carrier diffusion and traditional band-edge carrier transport (Fig. 1e, top), which are highly important for understanding the work mechanisms of two-dimensional optoelectronic devices based on TMDC monolayers^51^. It is worth noting that the hot-carrier diffusion in the C-exciton state of MoS_2_ monolayer not only possesses the free-carrier relaxation feature, but also exhibits an initial-exciton-density-dependent second-order recombination rate constant. The latter could reflect the initial excited-state Coulomb environment around the photogenerated C-excitons. This contrasts with the band-edge free-carrier relaxation behaviour, such as in organometallic halide perovskite films^46^, where the second-order recombination rate constant is invariant under different pump fluences.

In summary, our results establish a fundamental photophysical model in MoS_2_ monolayers by carefully analysing the excited-state relaxation processes, especially the hot-carrier cooling and initial extraction for the C-exciton state in the band nesting region. A two-body recombination mechanism for the hot-carrier relaxation in the C-exciton state is proposed. In addition, an unprecedented initial hot-carrier extraction efficiency of 80% for the C-exciton state at room temperature is further identified when we use graphene as the electron acceptor (scheme in Fig. 1f, top). This highly efficient initial charge separation in the vertical direction occurred among the band-edge states (the initial carrier extraction efficiency is 93% and 81% for the band-edge A- and B-exciton states, respectively) and all high-energy excited-states indicates the optimized fabrication route for the construction of all two-dimensional van der Waals heterostructure devices. The heterostructure also yields a twofold reduction of *E*_b_ in comparison with the pristine MoS_2_ monolayer (Fig. 1f, bottom). This further implies one approach to modulate the *E*_g_ of single TMDC monolayer crystals by locally overlapping with other dielectric screening materials. Further theoretical research such as time-dependent quantum calculation is necessary for a better understanding of many-body processes involving the hot-carrier generation, relaxation and initial extraction in these atomically thin two-dimensional heterostructures.

## Methods

### Sample preparation

MoS_2_ monolayers were grown by CVD on SiO_2_/Si substrates^27^. For the fabrication, the substrate was placed at the centre of the furnace, while the precursor powder (sulfur, 0.15 g) was placed in a quartz boat in a suitable upstream position. The smooth side of the SiO_2_/Si substrate was placed face down on a customized quartz half rod leaving a 2-μm gap between the substrate and the rod, and 0.05 g of MoO_3_ was put on the back of the substrate. The quartz tube was heated from room temperature to 650 °C with inlet of 50-sccm Ar, and when the temperature reached the maximum value, the Ar flow was adjusted to 10 sccm. The furnace was kept for 20 min at 650 °C for the growth of MoS_2_. Uniform MoS_2_ monolayers were obtained on the smooth side of the SiO_2_/Si substrate. For the MoS_2_ transfer^28^, the MoS_2_ film on Si wafer was first coated with a layer of PMMA (950 K, A3) by spin-coating (step 1: 500 r.p.m. for 10 s; step 2: 2,000 r.p.m. for 70 s), followed by annealing at 130 °C for 2 min. The PMMA at the edges of the SiO_2_/Si substrate was removed with a sharp blade to facilitate the following exfoliation of PMMA-capped MoS_2_ from substrate. Then, a NaOH (3 mol l^−1^) solution at 100 °C was used to exfoliate the PMMA-capped MoS_2_ from SiO_2_/Si. After that, the PMMA-supported MoS_2_ film was transferred to deionized (DI) water so as to remove the etchant and residues. Fresh quartz substrates with and without graphene were then used to fish out the PMMA-capped MoS_2_ film, followed by drying on a hot-plate (75 °C for 5 min and then 100 °C for 10 min). Hence, both MoS_2_ monolayer and MoS_2_ monolayer/graphene heterostructure samples were placed on quartz substrates. The PMMA was finally removed by acetone and the sample cleaned with isopropyl alcohol.

### Steady-state characterizations

The optical imaging was performed in a Nikon Eclipse LV 100D system, and the atomic force microscopy imaging was performed by a BRUKER Dimension FastScan system using the tapping mode. The Raman/PL spectra and mapping were collected in a confocal system (The Alpha 300R). The wavelength of the excitation laser was 532 nm with a spot size of approximately 0.5 mm. The steady-state PL spectra and mapping were obtained with a 600 grooves mm^−1^ grating, while a 1,800 grooves mm^−1^ grating was used to get detailed line shapes of the Raman band. The steady-state absorption spectra were measured using a Shimadzu UV-2550 spectrophotometer. Scanning tunnelling microscopy (STM) measurements were carried out in an ultra-high vacuum system housing an Omicron LT-STM interfaced to a Nanonis controller. The sample was degassed overnight at 350 °C before STM/STS analysis was performed, and kept at 77 K during the measurements. For d*I*/d*V* spectra, the tunnelling current was obtained by a lock-in amplifier, with a modulation of 625 Hz and 40 mV. Note that the bias voltage (*V*_tip_) is applied on the STM tip; hence negative values correspond to the conduction band and positive values to the valence band.

### Femtosecond broadband transient absorption setup

In the TA setup for both transmission and reflection modes, a mode-locked Ti:sapphire laser/amplifier system (Solstice, Spectra-Physics) was used (800 nm wavelength, 1.5-mJ pulse energy, 100 fs pulse width, 250 Hz repetition rate). The low frequency was set to allow matching of the signal with the collection system. The output of the amplifier was split into two parts, with the stronger beam used to generate the desired excitation light. For 600 and 650 nm excitation, a TOPAS system was adopted, while 400 nm excitation was directly doubled from 800 nm laser pulses. The broad-band white-light probe pulses from 400 to 850 nm generated from 2 mm thick water. The TA data were collected by a fibre-coupled spectrometer connected to a computer. The group velocity dispersion of the transient spectra was compensated by a chirp program. All the measurements were performed at room temperature.

### Theoretical calculations

We performed the calculations using the DFT as implemented in the VASP codes. The exchange correlation energy was described by the generalized gradient approximation, in the scheme proposed by Perdew, Burke and Ernzerhof (PBE) (refs 52, 53). A unit cell with vacuum region of 16 Å was used for the calculation, and a 9 × 9 × 1 Monkhorst-Pack mesh grid was used to sample the Brillouin zone. The cutoff energy of the plane wave basis was 520 eV. All atoms were relaxed until the Hellman-Feynman forces on individual atoms were less than 0.02 eV Å^−1^. The effect of spin–orbit coupling was also included. Although the PBE calculation could underestimate MoS_2_'s bandgap^54^, the resulting profile of energy band is expected to be correct.

### Data availability

The data that support the findings of this study are available from the corresponding author on request.

## Additional information

**How to cite this article:** Wang, L. *et al*. Slow cooling and efficient extraction of C-exciton hot carriers in MoS_2_ monolayer. *Nat. Commun.* **8,** 13906 doi: 10.1038/ncomms13906 (2017).

**Publisher's note:** Springer Nature remains neutral with regard to jurisdictional claims in published maps and institutional affiliations.

## Supplementary Material

Supplementary Information Supplementary Figures, Supplementary Table, Supplementary Notes, Supplementary References

## References

[b1] Gabor N. M. . Hot carrier assisted intrinsic photoresponse in graphene. Science 334, 648–652 (2011). 21979935 10.1126/science.1211384

[b2] Brongersma M. L., Halas N. J. & Nordlander P. Plasmon-induced hot carrier science and technology. Nat. Nanotechnol. 10, 25–34 (2015). 25559968 10.1038/nnano.2014.311

[b3] Shockly W. & Queisser H. J. Detailed balance limit of efficiency of p-n junction solar cells. J. Appl. Phys. 32, 510–519 (1961).

[b4] Ross R. T. & Nozik A. J. Efficiency of hot-carrier solar energy converters. J. Appl. Phys. 53, 3813–3818 (1982).

[b5] Nozik A. J. Spectroscopy and hot electron relaxation dynamics in semiconductor quantum well and quantum dots. Annu. Rev. Phys. Chem. 52, 193–231 (2001). 11326064 10.1146/annurev.physchem.52.1.193

[b6] Wang Y. F. . Electron extraction dynamics in CdSe and CdSe/CdS/ZnS quantum dots adsorbed with methyl viologen. J. Phys. Chem. C 118, 17240–17246 (2014).

[b7] Tisdale W. A. . Hot-electron transfer from semiconductor nanocrystals. Science 328, 1543–1547 (2010). 20558714 10.1126/science.1185509

[b8] Efros A. L., Kharchenko V. A. & Rosen M. Breaking the phonon bottleneck in nanometer quantum dots: role of Auger-like processes. Solid State Commun. 93, 281–284 (1995).

[b9] Cooney R. R., Sewall S. L., Anderson K. E. H., Dias E. A. & Kambhampati P. Breaking the phonon bottleneck for holes in semiconductor quantum dots. Phys. Rev. Lett. 98, 117403 (2007).

[b10] Pandey A. & Guyot-Sionnest P. Slow electron cooling in colloidal quantum dots. Science 322, 929–932 (2008). 18988849 10.1126/science.1159832

[b11] Xu M. S., Liang T., Shi M. M. & Chen H. Z. Graphene-like two-dimensional materials. Chem. Rev. 113, 3766–3798 (2013). 23286380 10.1021/cr300263a

[b12] Chhowalla M. . The chemistry of two-dimensional layered transition metal dichalcogenide nanosheets. Nat. Chem. 5, 263–275 (2013). 23511414 10.1038/nchem.1589

[b13] Bonaccorso F. . Graphene, related two-dimensional crystals, and hybrid systems for energy conversion and storage. Science 347, 1246501 (2015). 25554791 10.1126/science.1246501

[b14] Xia F. N., Wang H., Xiao D., Dubey M. & Ramasubramaniam A. Two-dimensional material nanophotonics. Nat. Photon. 8, 899–907 (2014).

[b15] Wang Q. H., Kalantar-Zadeh K., Kis A., Coleman J. N. & Strano M. S. Electronics and optoelectronics of two-dimensional transition metal dichalcogenides. Nat. Nanotechnol. 7, 699–712 (2012). 23132225 10.1038/nnano.2012.193

[b16] Eda G. & Maier S. A. Two-dimensional crystals: managing light for optoelectronics. ACS Nano 7, 5660–5665 (2013). 23834654 10.1021/nn403159y

[b17] Memaran S. . Pronounced photovoltaic response from multilayered transition-metal dichalcogenides PN-junctions. Nano Lett. 15, 7532–7538 (2015). 26513598 10.1021/acs.nanolett.5b03265

[b18] Carvalho A., Ribeiro R. M. & Castro Neto A. H. Band nesting and the optical response of two-dimensional semiconducting transition metal dichalcogenides. Phys. Rev. B 88, 115205 (2013).

[b19] Kozawa D. . Photocarrier relaxation pathway in two-dimensional semiconducting transition metal dichalcogenides. Nat. Commun. 5, 4543 (2014). 25072556 10.1038/ncomms5543

[b20] Kumar R., Verzhbitskiy I. & Eda G. Strong optical absorption and photocarrier relaxation in 2-D semiconductors. IEEE J. Quantum Electron. 51, 0600206 (2015).

[b21] Ugeda M. M. . Giant bandgap renormalization and excitonic effects in a monolayer transition metal dichalcogenide semiconductor. Nat. Mater. 13, 1091–1095 (2014). 25173579 10.1038/nmat4061

[b22] Mak K. F., Lee C. G., Hone J., Shan J. & Heinz T. F. Atomically thin MoS_2_: a new direct-gap semiconductor. Phys. Rev. Lett. 105, 136805 (2010). 21230799 10.1103/PhysRevLett.105.136805

[b23] Splendiani A. . Emerging photoluminescence in monolayer MoS_2_. Nano Lett. 10, 1271–1275 (2010). 20229981 10.1021/nl903868w

[b24] Kumar N., He J. Q., He D. W., Wang Y. S. & Zhao H. Charge carrier dynamics in bulk MoS_2_ crystal studied by transient absorption microscopy. J. Appl. Phys. 113, 133702 (2013).

[b25] Borzda T. . Charge photogeneration in few-layer MoS_2_. Adv. Funct. Mater. 25, 3351–3358 (2015).

[b26] Pogna E. A. A. . Photo-induced bandgap renormalization governs the ultrafast response of single-layer MoS_2_. ACS Nano 10, 1182–1188 (2016). 26691058 10.1021/acsnano.5b06488

[b27] Lee Y. H. . Synthesis of large-area MoS_2_ atomic layers with chemical vapor deposition. Adv. Mater. 24, 2320–2325 (2012). 22467187 10.1002/adma.201104798

[b28] Lin Y. C. . Wafer-scale MoS_2_ thin layers prepared by MoO_3_ sulfurization. Nanoscale 4, 6637–6641 (2012). 22983609 10.1039/c2nr31833d

[b29] Mertens J. . Excitons in a mirror: formation of “optical bilayers” using MoS_2_ monolayers on gold substrates. Appl. Phys. Lett. 104, 191105 (2014).

[b30] Huang Y. L. . Bandgap tenability at single-layer molybdenum disulphide grain boundaries. Nat. Commun. 6, 6298 (2015). 25687991 10.1038/ncomms7298

[b31] Cheiwchanchamnangij T. & Lambrecht W. R. L. Quasiparticle band structure calculation of monolayer, bilayer, and bulk MoS_2_. Phys. Rev. B 85, 205302 (2012).

[b32] Ramasubramaniam A. Large excitonic effects in monolayers of molybdenum and tungsten dichalcogenides. Phys. Rev. B 86, 115409 (2012).

[b33] Shi H. L., Pan H., Zhang Y. W. & Yakobson B. I. Quasiparticle band structures and optical properties of strained monolayer MoS_2_ and WS_2_. Phys. Rev. B 87, 155304 (2013).

[b34] Molina-Sánchez A., Sangalli D., Hummer K., Marini A. & Wirtz L. Effect of spin-orbit interaction on the optical spectra of single-layer, double-layer, and bulk MoS_2_. Phys. Rev. B 88, 045412 (2013).

[b35] Qiu D. Y., da Jornada F. H. & Louie S. G. Optical spectrum of MoS_2_: many-body effects and diversity of exciton states. Phys. Rev. Lett. 111, 216805 (2013). 24313514 10.1103/PhysRevLett.111.216805

[b36] Wang L. . Direct observation of quantum-confined graphene-like states and novel hybrid states in graphene oxide by transient spectroscopy. Adv. Mater. 25, 6529–6545 (2013). 10.1002/adma.201302927 24030902

[b37] Wang L. . Ultrafast optical spectroscopy of surface-modified silicon quantum dots: unraveling the underlying mechanism of the ultrabright and color-tunable photoluminescence. Light Sci. Appl. 4, e245 (2015).

[b38] Sim S. W. . Exciton dynamics in atomically thin MoS_2_: interexcitonic interaction and broadening kinetics. Phys. Rev. B 88, 075434 (2013).

[b39] Moody G. . Intrinsic homogeneous linewidth and broadening mechanisms of excitons in monolayer transition metal dichalcogenides. Nat. Commun. 6, 8315 (2015). 26382305 10.1038/ncomms9315 PMC4595717

[b40] Moody G., Schaibley J. & Xu X. D. Exciton dynamics in monolayer transition metal dichalcogenides. J. Opt. Soc. Am. B 33, C39–C49 (2016). 10.1364/JOSAB.33.000C39 PMC5590662 28890600

[b41] Lakowicz J. R. Principles of Fluorescence Spectroscopy 3rd edn Springer (2006).

[b42] Sun D. Z. . Observation of rapid exciton-exciton annihilation in monolayer molybdenum disulfide. Nano Lett. 14, 5625–5629 (2014). 25171389 10.1021/nl5021975

[b43] Kumar N. . Exciton-exciton annihilation in MoSe_2_ monolayers. Phys. Rev. B 89, 125427 (2014).

[b44] Docherty C. J. . Ultrafast transient terahertz conductivity of monolayer MoS_2_ and WSe_2_ grown by chemical vapor deposition. ACS Nano 8, 11147–11153 (2014). 25347405 10.1021/nn5034746

[b45] Chernikov A., Ruppert C., Hill H. M., Rigosi A. F. & Heinz T. F. Population inversion and giant bandgap renormalization in atomically thin WS_2_ layers. Nat. Photon. 9, 466–470 (2015).

[b46] Manser J. S. & Kamat P. V. Band filling with free charge carriers in organometal halide perovskites. Nat. Photon. 8, 737–743 (2014).

[b47] Mueller M. L., Yan X., Dragnea B. & Li L. S. Slow hot-carrier relaxation in colloidal graphene quantum dots. Nano Lett. 11, 56–60 (2011). 21126052 10.1021/nl102712x

[b48] Klimov V. I. Spectral and dynamical properties of multiexcitons in secmiconductor nanocrystals. Annu. Rev. Phys. Chem. 58, 635–673 (2007). 17163837 10.1146/annurev.physchem.58.032806.104537

[b49] He J. Q. . Electron transfer and coupling in graphene-tungsten disulfide van der Waals heterostructures. Nat. Commun. 5, 5622 (2014). 25421098 10.1038/ncomms6622

[b50] Ceballos F., Bellus M. Z., Chiu H. Y. & Zhao H. Ultrafast charge separation and indirect exciton formation in a MoS_2_-WSe_2_ van der Waals heterostructures. ACS Nano 8, 12717–12724 (2014). 25402669 10.1021/nn505736z

[b51] Lee C. H. . Atomically thin p-n junctions with van der Waals heterointerfaces. Nat. Nanotechnol. 9, 676–681 (2014). 25108809 10.1038/nnano.2014.150

[b52] Perdew J. P., Burke K. & Ernzerhof M. Generalized gradient approximation made simple. Phys. Rev. Lett. 77, 3865–3868 (1996). 10062328 10.1103/PhysRevLett.77.3865

[b53] Wang D. . Determination of formation and ionization energies of charge defects in two-dimensional materials. Phys. Rev. Lett. 114, 196801 (2015). 26024189 10.1103/PhysRevLett.114.196801

[b54] Komsa H. P. & Krasheninnikov A. V. Effects of confinement and environment on the electronic structure and exciton binding energy of MoS_2_ from first principles. Phys. Rev. B 86, 241201(R) (2012).

