# Supplementary material for: Slow cooling and efficient extraction of C-exciton hot carriers in MoS2 monolayer
Source: Nat Commun. 2017 Jan 5;8:13906. doi: 10.1038/ncomms13906 (PMC5227064; doi:10.1038/ncomms13906)
Supplement: Supplementary Information — Supplementary Figures, Supplementary Table, Supplementary Notes, Supplementary References [file ncomms13906-s1.pdf]

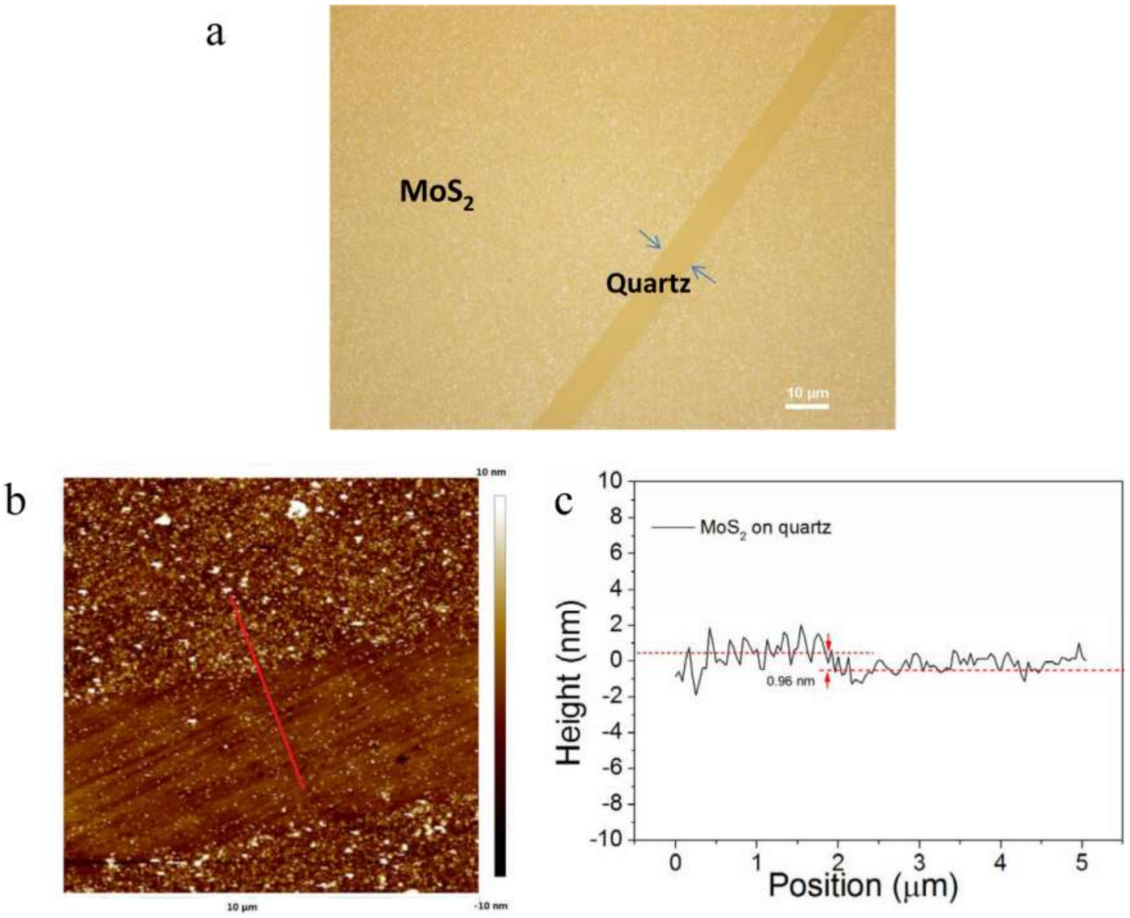

Supplementary Figure 1. **a**, Optical microscope image of MoS_2 monolayers on quartz under 100× objective; **b**, AFM height profile of MoS_2 monolayers on quartz; **c**, Cross-sectional profile along the red line in **b**, which is consistent with the reported thickness of 0.6–0.9 nm for MoS_2 monolayers on substrates[1].

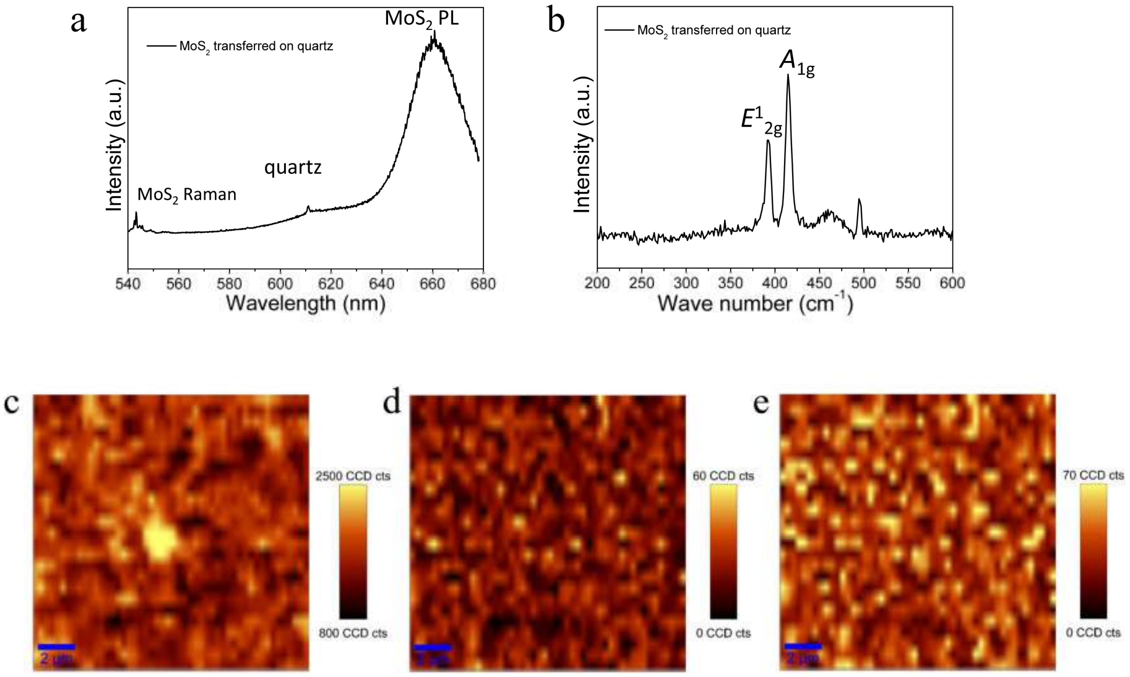

Supplementary Figure 2. **a**, Steady-state PL spectrum of MoS_2 monolayers on quartz; **b**, Raman spectrum of MoS_2 monolayers on quartz; **c**, PL mapping (640–680 nm) of MoS_2 monolayers on quartz; **d**, E^1_{2g} mapping (388–399 cm^{-1}) of MoS_2 monolayers on quartz; **e**, A_{1g} mapping (409–421 cm^{-1}) of MoS_2 monolayers on quartz.

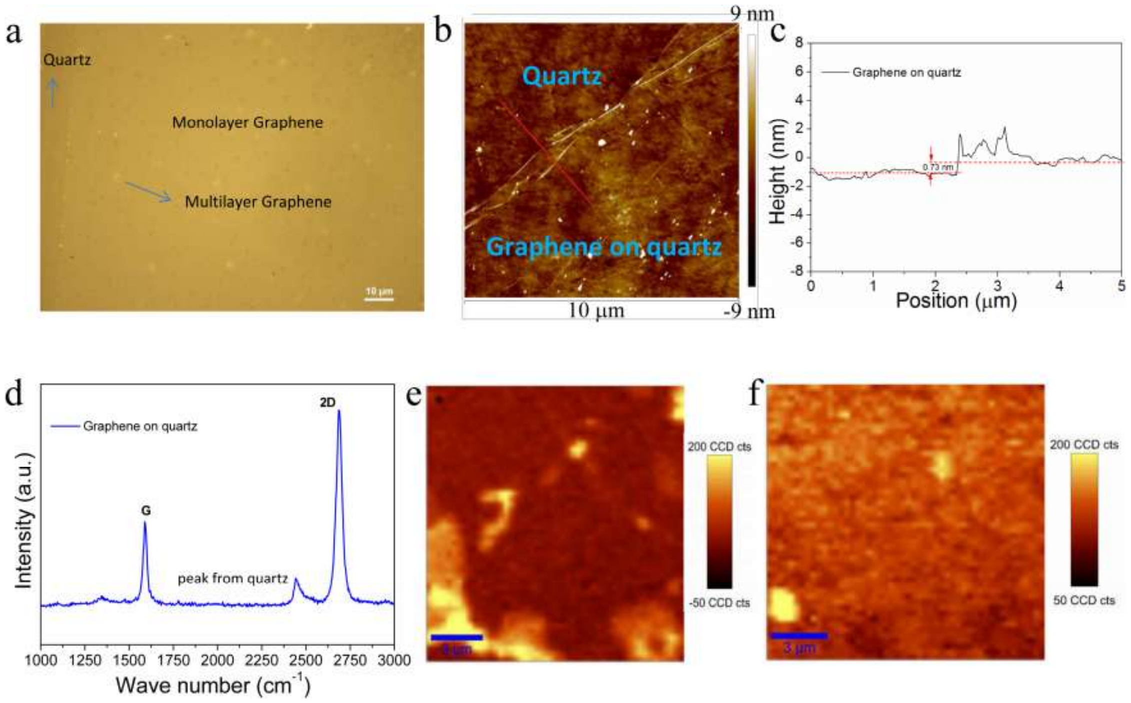

Supplementary Figure 3. **a**, Optical microscope image of graphene on quartz under 100× objective; **b**, AFM height profile of graphene on quartz; **c**, Cross-sectional profile along the red line in **b**. **d**, Raman spectrum of graphene on quartz; **e**, G peak (1562–1640 cm^{-1}) mapping of graphene on quartz; **f**, 2D peak (2638–2739 cm^{-1}) mapping of graphene on quartz.

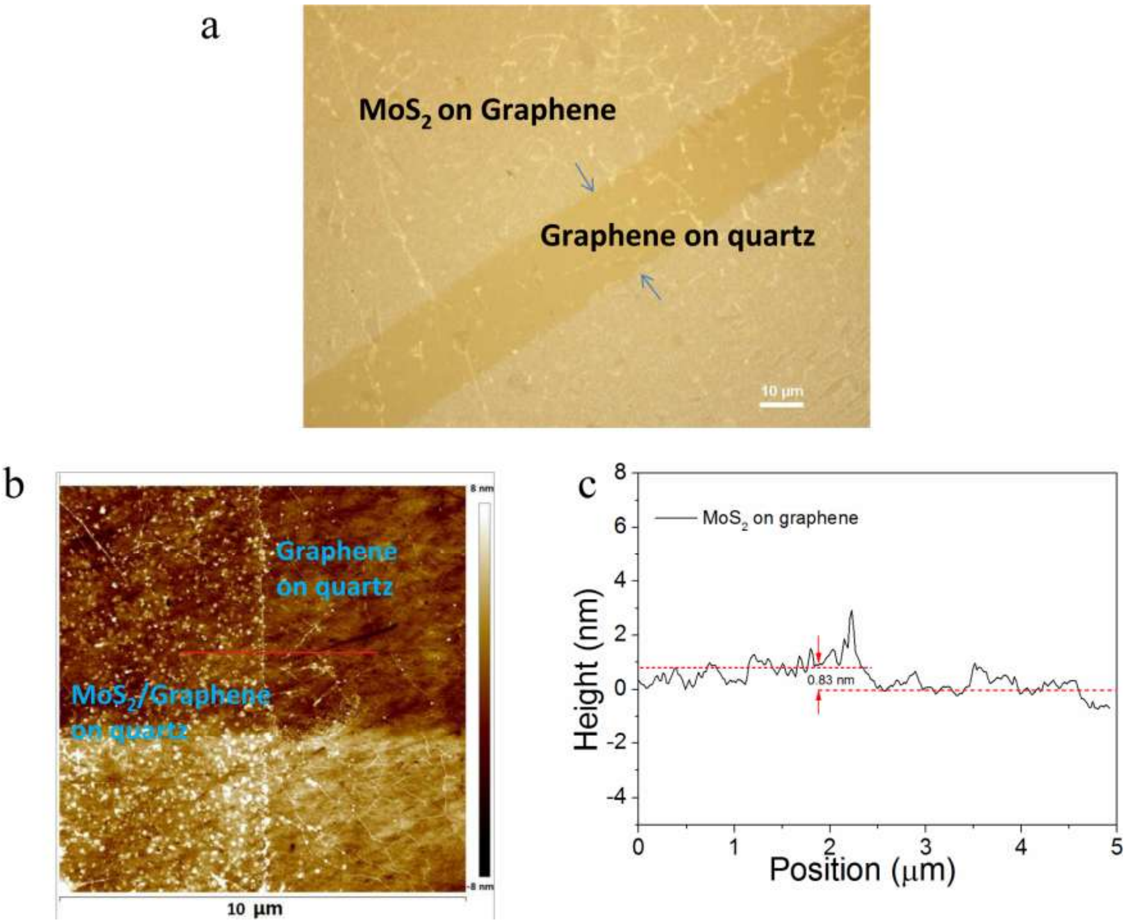

Supplementary Figure 4. a, Optical microscope image of MoS_2 monolayer/graphene on quartz under 100× objective; **b**, AFM height profile of MoS_2 monolayer/graphene on quartz; **c**, Cross-sectional profile along the red line in **b**.

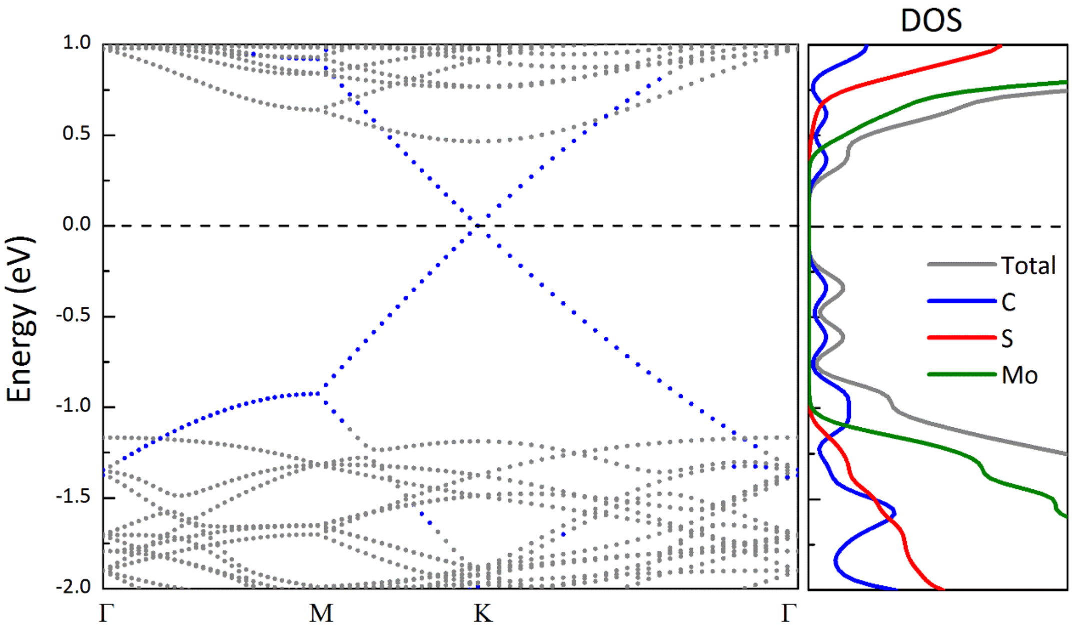

Supplementary Figure 5. DFT calculation of the projected band structure (left) and density of states (right) of MoS_2-graphene heterostructure. The blue dots (left figure) represent the contribution of carbon atoms in graphene.

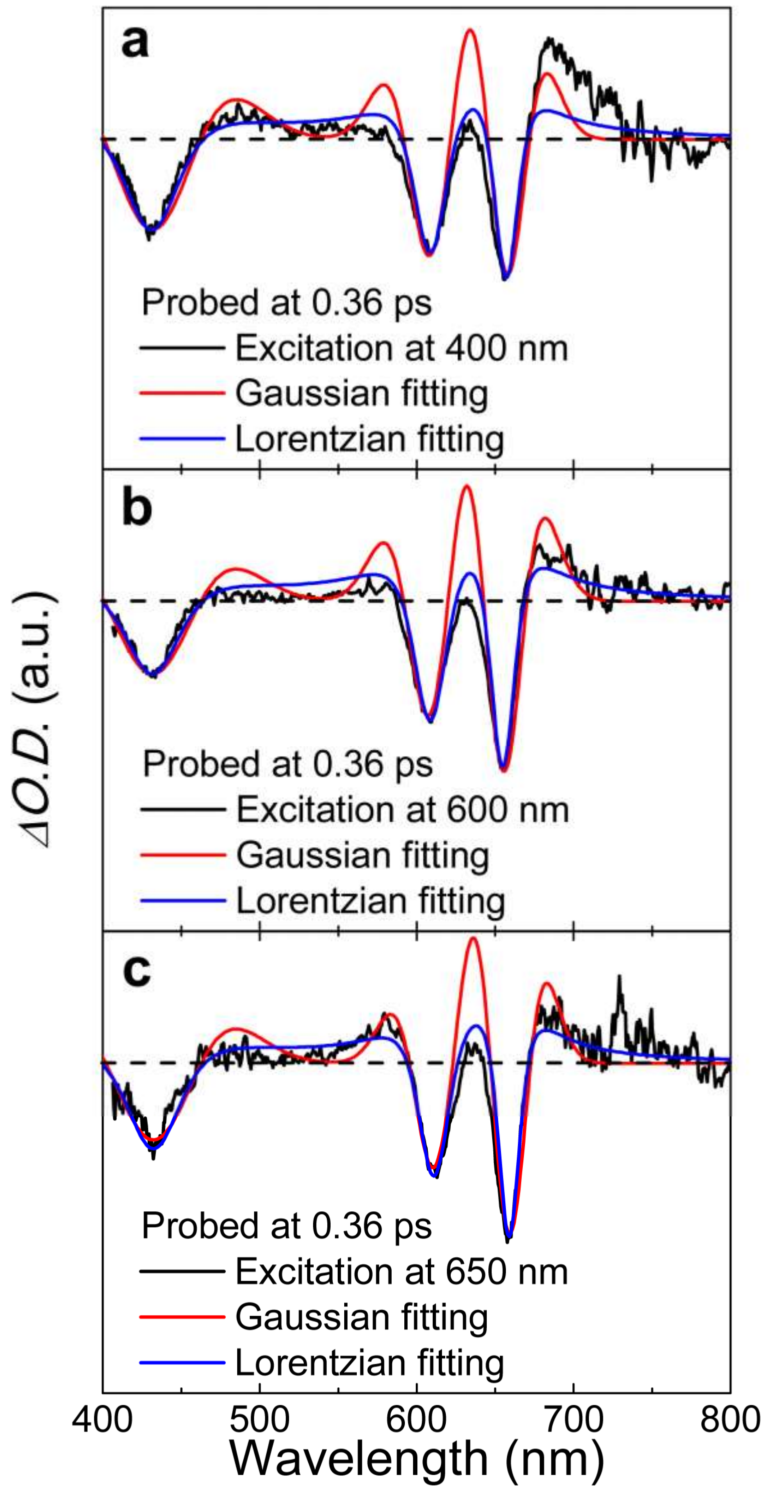
a
Probed at 0.36 ps
Excitation at 400 nm
Gaussian fitting
Lorentzian fitting
b
Probed at 0.36 ps
Excitation at 600 nm
Gaussian fitting
Lorentzian fitting
c
Probed at 0.36 ps
Excitation at 650 nm
Gaussian fitting
Lorentzian fitting
ΔO.D. (a.u.)
400
500
600
700
800
Wavelength (nm)

Supplementary Figure 6. Gaussian and Lorentzian fitting of initial transient absorption spectra (probed at 0.36 ps) of MoS_2 monolayer under (a) 400 nm excitation, (b) 600 nm excitation and (c) 650 nm excitation (initial exciton density of ~1.2 × 10^{12} cm^{-2}). The Lorentzian fitting proves to be the best option and could be used to estimate the contributions of peak shift and broadening. The remainder of positive signals could belong to excited-state absorption.

Supplementary Note 1. Clearly, if we only consider the peak shift and broadening of the A/B/C-exciton states, the positive signals observed in our transient absorption experiments cannot be reproduced. Furthermore, for higher initial exciton densities (> 2 × 10^{12} cm^{-2}), the difference between the Gaussian/Lorentzian fitting results and the experiment is more remarkable. For a reliable universal model on the recovery dynamics, crucial factors such as transient photoluminescence (PL, radiative recombination processes), excited state (Rydberg series) relaxation of band-edge excitons, and additional excited-state absorption signals should be considered, which are discussed later in Supplementary Figure 10 and Supplementary Note 4.

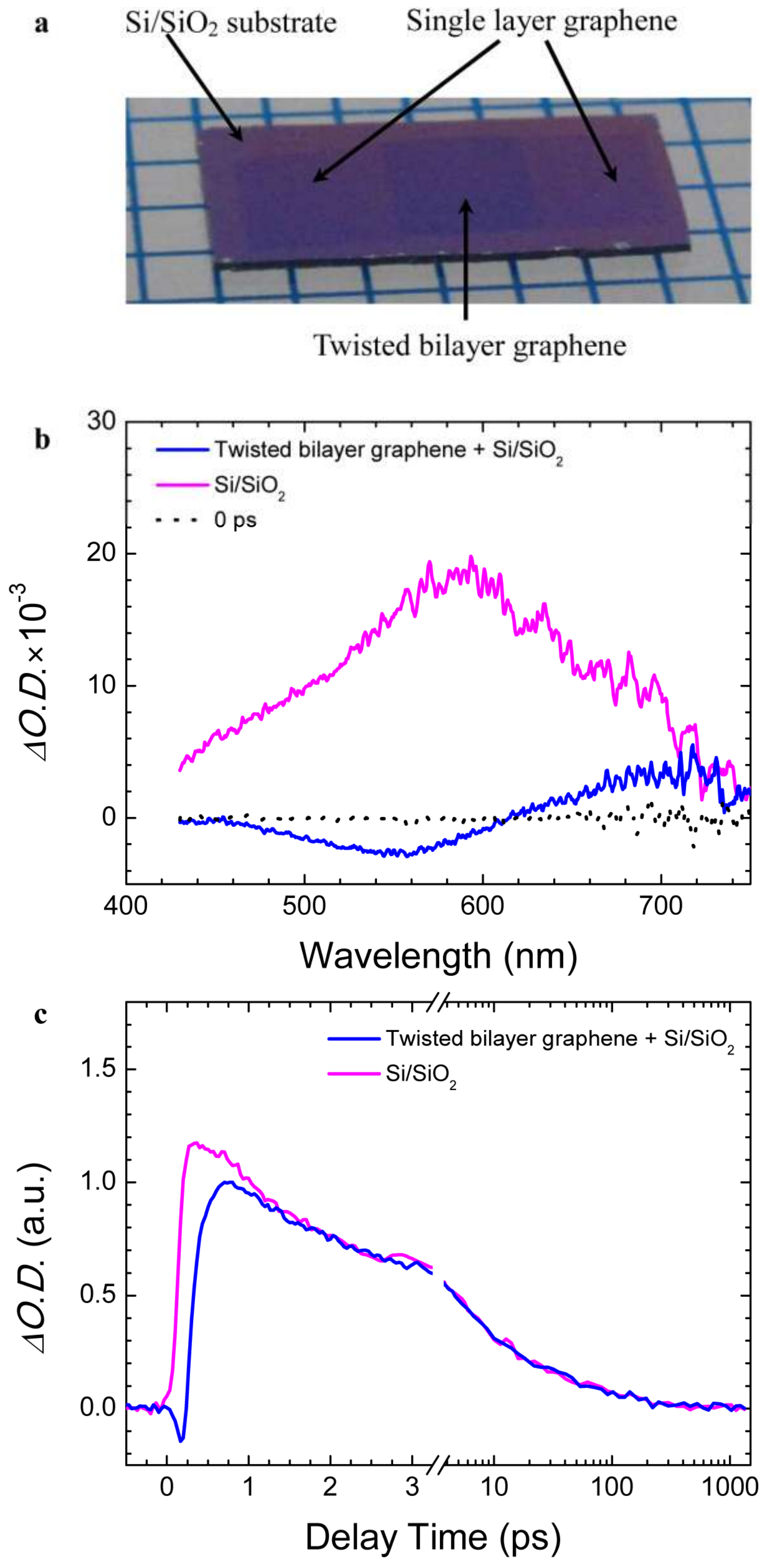
a
Si/SiO2 substrate
Single layer graphene
Twisted bilayer graphene
b
Twisted bilayer graphene + Si/SiO2
Si/SiO2
0 ps
ΔO.D.×10-3
Wavelength (nm)
c
Twisted bilayer graphene + Si/SiO2
Si/SiO2
ΔO.D. (a.u.)
Delay Time (ps)

Supplementary Figure 7. **a**, Photography of the twisted bilayer graphene on Si/SiO_2 studied here. **b**, Transient absorption spectra of the twisted bilayer graphene (blue line), and pure Si/SiO_2 substrate (magenta line) probed at 0.2 ps under 400 nm excitation (300 μJ cm^{-2}). The dot line indicates the spectrum probed at 0 ps. **c**, The dynamics probed at 580 nm for the twisted bilayer graphene, and Si/SiO_2 substrate.

Supplementary Note 2. Reflection mode TA experiments were performed on twisted bilayer graphene, fabricated by overlapping two pieces of graphene flakes (grown by CVD method) on a Si/SiO_2 (300 nm) substrate[3]. In Supplementary Figure 7b, for the TA spectra of the twisted bilayer graphene, compared to the pure Si/SiO_2 substrate, there is an additional negative signal. By subtracting the contribution from the substrate, the spectrum presented in the inset of Supplementary Figure 8b is obtained. A large negative signal peaked at 580 nm (2.14 eV) can be found for the pure twisted bilayer. According to the observed energy gaps for the parallel bands (which represent the state of Van Hove singularity, VHS), $E_{VHS} = E_0 \sin(3\theta)$ with $E_0 = 3.9$ eV[4] in the three measured samples, and a twist angle around 11°~12° between the two graphene layers in the twisted bilayer graphene can be deduced, which is consistent with previous reports[5,6]. The characteristic dynamics probed at 580 nm are shown in the Supplementary Figure 7c. The long-lifetime components corresponding to the Si/SiO_2 substrate are normalized to coincide with each

other. By subtracting this substrate contribution from the original dynamics of the twisted bilayer graphene, assuming no interaction between the both, we can extract the pure dynamics of the parallel bands (the VHS state, double difference signal, *ΔΔO.D.*) as shown in Supplementary Figure 8b.

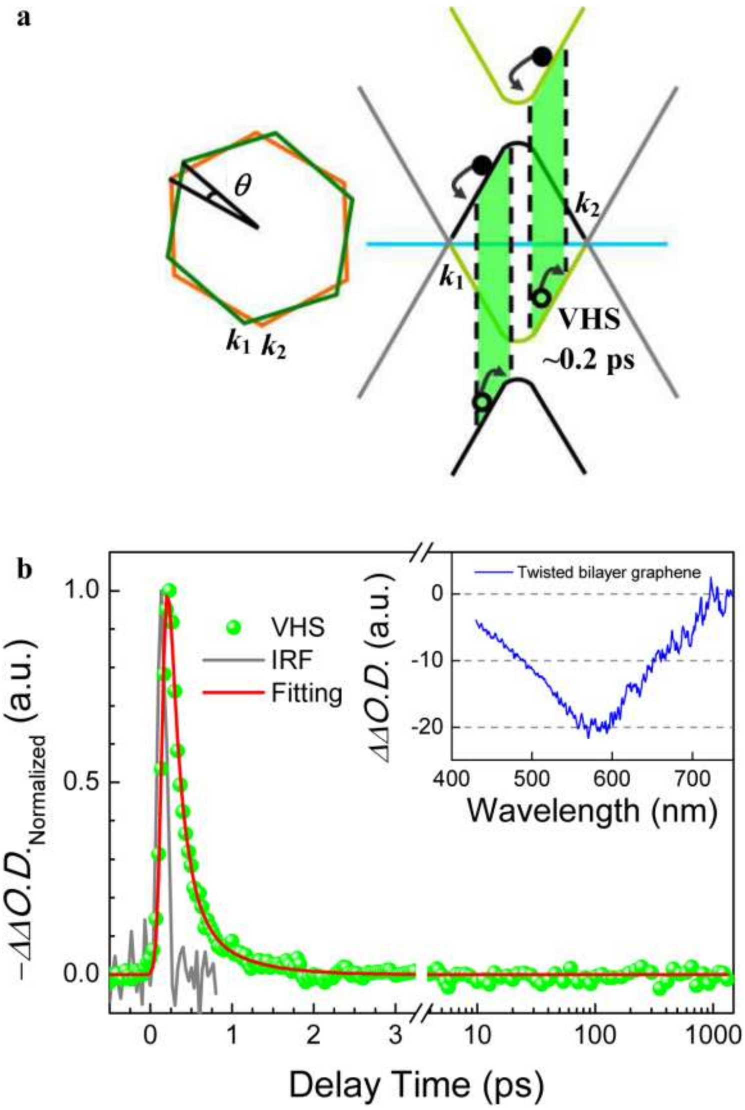

Supplementary Figure 8. **a**, Illustration of the Brillouin zones rotated by θ and corresponding energy band structure in the twisted bilayer graphene. k_1 and k_2 represent each of graphene layers. Black and lime arrows indicate the carrier recombination in parallel bands (the VHS state). **b**, Normalized dynamics of the VHS state (double difference signal, $\Delta\Delta O.D.$) probed at 580 nm for the twisted bilayer graphene. Solid green spheres represent the obtained data of the VHS state in the twisted bilayer graphene. Red

line is the fitting curve. Two-exponential fitting gives an average decay lifetime of 0.21 ps [τ_1 = 0.17 ps (97%), and τ_2 = 1.50 ps (3%)]. The gray line is the instrument response function (IRF). Inset shows the obtained TA spectrum at 0.2 ps for the pure twisted bilayer graphene.

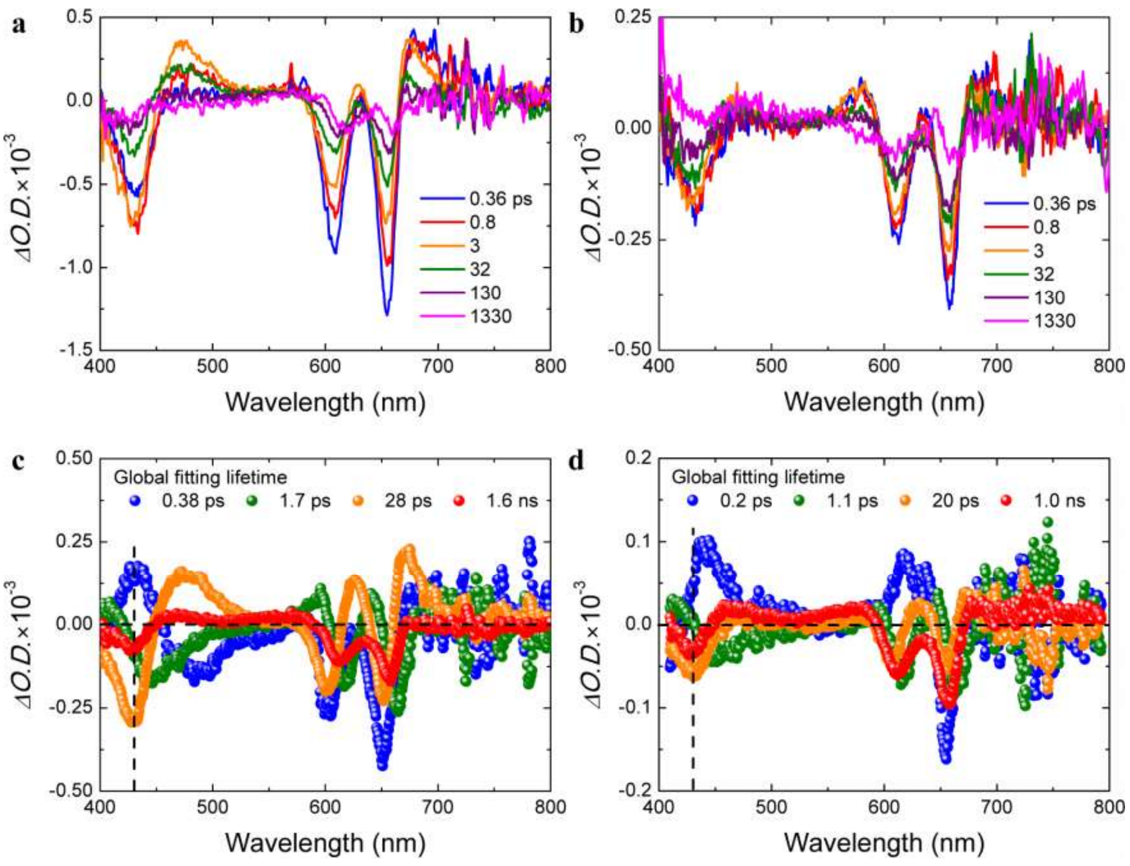

Supplementary Figure 9. a, Transient absorption spectra of MoS_2 monolayers probed at different delay times under 600 nm excitation (initial exciton density of 1.27×10^{12} cm^{-2}). **b,** Transient absorption spectra of MoS_2 monolayers probed at different delay times under 650 nm excitation (initial exciton density of 1.13×10^{12} cm^{-2}). **c**, Global analysis for the transient absorption data in panel **a**. **d**, Global analysis for the transient absorption data in panel **b**.

Supplementary Note 3. In the global analysis of band-edge excitations (Supplementary Figure 9c,d) and 400 nm excitation (Fig. 2b) TA experiments, the main difference comes

from the shortest lifetime components. For the 600 nm excitation case (Supplementary Figure 9c), there is a positive excited-state absorption peak in the lifetime component of 0.38 ps, which is located at the same spectral position of the C-exciton state. What is more, this kind of positive excited-state absorption peak also appears in the lifetime component of 0.2 ps for the 650 nm excitation case (Supplementary Figuer 9d), although a little red-shifted. In contrast, in the global analysis of the 400 nm excitation TA experiment (Fig. 2b), there is no positive signal around the position of the C-exciton state in the lifetime component of 0.27 ps.

Since band-edge excitations cannot directly generate the high-energy C-excitons, this positive excited-state absorption peak could be related to the formation of C-excitons. There are two possible mechanisms for the formation of C-exciton under band-edge excitations. One is directly tunneling from the band-edge A-/B-exciton state to the C-exciton state. However, due to the direct bandgap energy band structure in monolayer MoS_2, this directly intervalley tunneling may not be as efficient as that in the bulk case[7]. The other mechanism, which is more feasible, involves photogenerated band-edge excitons jumping firstly into the high excited states by the exciton-exciton annihilation, and then fast tunneling to the C-exciton state by an energy state hybridization[8]. Furthermore, since the excited states of B-exciton are always slightly higher than those of A-exciton in energy, our global analysis for the positive excited-state absorption peak in the high-energy region in Supplementary Figure 9c,d may also reflect this trend. In

summary, regarding our proposed upconversion process, it combines the two following processes in the following way: firstly, the band-edge exciton-exciton annihilation effect, which produces high-energy excited-state excitons of band-edge excitons. From our global analysis on the band-edge excitation cases, we deduce that the highest excited-states of band-edge excitons are occupied due to the exciton-exciton annihilation effect; and secondly, a tunneling process, which is indeed feasible according to the theoretically predicted hybridization between these excited states and the *C*-exciton state. In this context, the large energy difference between the *C*-excitons and band-edge excitons can be overcome even at room temperature.

There are also some differences between 600 nm and 650 nm excitations. For example, there is an additional positive excited-state absorption peak in the shortest lifetime component of 0.2 ps in the 650 nm excitation case that matches the spectral position of B-exciton well. This implies a transient formation of the B-exciton, accompanied by a decrease of A-exciton populations. Consistent with this, there is a lack of a fast decay component for the dynamics of the B-exciton in the 650 nm excitation case, compared to the dynamics of the A-exciton. In addition, the lifetime component of 1.1 ps in this excitation regime represents no contribution to the C-exciton state. The different distributions for each lifetime component in the band-edge and 400 nm excitation cases lead to an average lifetime of 350 ± 50 ps for the C-exciton state, which is still longer than the average lifetime of 310 ± 30 ps for the A-exciton state.

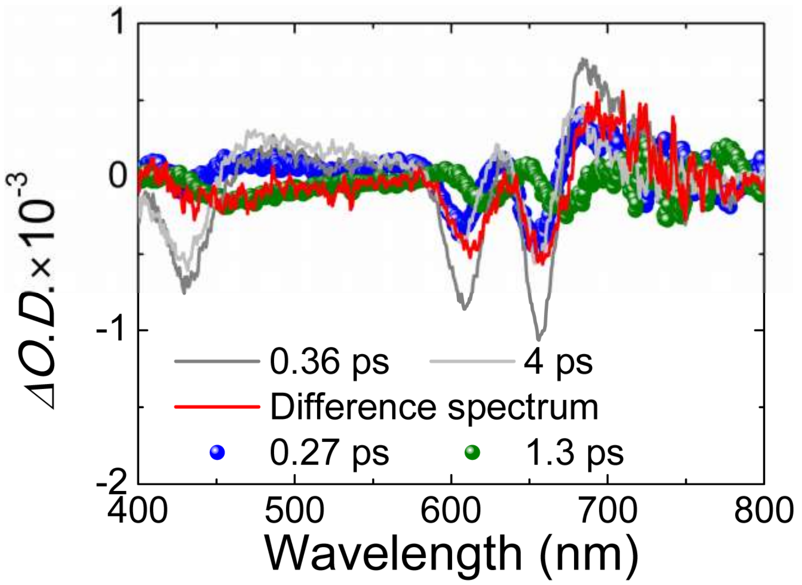

Supplementary Figure 10. Difference spectrum (red line) between the transient absorption spectra of MoS_2 monolayers probed at 0.36 ps (gray line) and 4 ps (light gray line) under 400 nm excitation (initial exciton density of 1.28×10^{12} cm^{-2}), indicating changes within the first 4 ps. Blue and green balls represent the 0.27 ps and 1.3 ps lifetime components obtained by global analysis, respectively.

Supplementary Note 4. Concerning negative signals in our transient absorption data, the two following possible contributions should be considered: firstly, the contribution of transient PL (stimulated emission) to the shape of transient absorption spectra at the band edge (600–700 nm) due to the direct bandgap structure of the MoS_2 monolayer; and secondly, the contribution of excited states (Rydberg series) of band-edge excitons to the shape of transient absorption spectra at 500–600 nm. We note that since both contributions are time-dependent, once the excited state (Rydberg series) of band-edge

excitons relaxes and radiative recombination processes finish, these contributions would vanish.

To find which transient species disappear during the first 4 ps, in Fig. S10 we subtract the transient absorption spectrum at 4 ps from that at 0.36 ps. We observe that the two negative narrow peaks, only slightly red-shifted relative to the band-edge exciton states wavelengths, could represent the disappearing of the MoS_2 monolayer's PL. At the same time, the broad band ranging from 430 nm to 560 nm could be attributed to a series of predicted and experimentally observed excited states (Rydberg series) of band-edge excitons covering that spectral region[8–11]. We note that since the relaxation dynamics of these excited states (1.3 ps lifetime component in Supplementary Figure 10) strongly overlap with other positive signals such as the 0.27 ps lifetime component, the global analysis results in a very useful method to determine different underlying physical processes.

Finally, we mention that the set of excited states at 2.60 eV predicted by S. G. Louie et al.[8], which hybridizes slightly with the excitons forming peak C (2.80 eV), matches well with our global analysis showing excited states of band-edge excitons at ~2.54 eV, close to the C-exciton state (2.88 eV). This supports our hypothesis of a fast tunneling mechanism from these excited states to the C-exciton state.

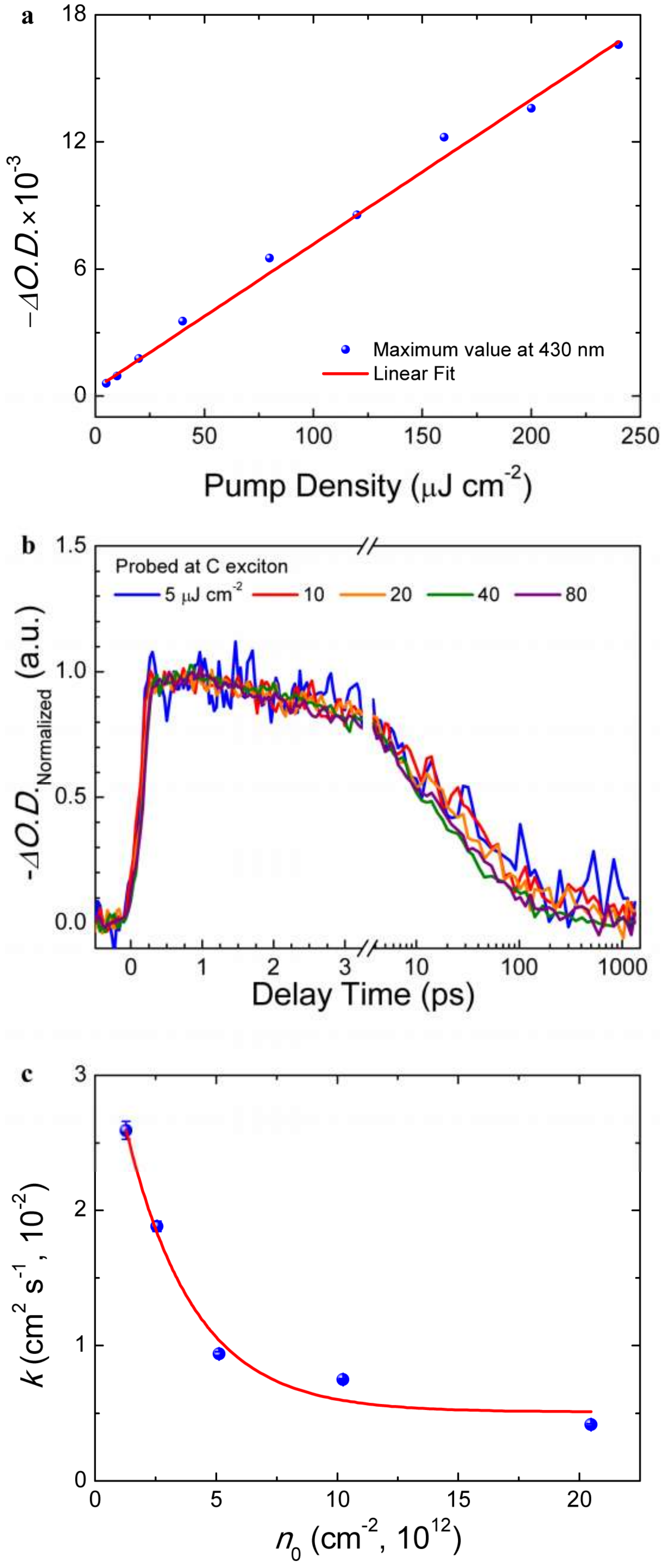
a
Maximum value at 430 nm
Linear Fit
$-\Delta O.D.\times10^{-3}$
Pump Density (μJ cm^{-2})
b
Probed at C exciton
5 μJ cm^{-2}
10
20
40
80
$-\Delta O.D._{Normalized}$ (a.u.)
Delay Time (ps)
c
k (cm^2 s^{-1}, 10^{-2})
n_0 (cm^{-2}, 10^{12})

Supplementary Figure 11. a, Linear fitting of the signal amplitude of the C-exciton state at 430 nm for different pump densities. **b**, Normalized decay dynamics of the C-exciton state under different excitation power densities. **c**, Second-order recombination rate constant of MoS_2 monolayer as a function of initial exciton density. The red solid line in panel **c** shows a fit using the phenomenological expression $k = \beta \exp(-n_0 / \delta) + \gamma$, where δ, β, and γ are the fitting parameters. This initial-exciton-density-dependent second-order recombination rate constant could be one of hot-carrier relaxation features in the *C*-exciton state of MoS_2 monolayer. By multiplying this value of $(3.8 \pm 0.2) \times 10^{-2}$ $cm^2\ s^{-1}$ obtained in the main text by the ideal thickness of MoS_2 monolayer (0.6–0.7 nm, 0.65 nm in average), we calculate a second-order recombination rate constant of $(2.5 \pm 0.2) \times 10^{-9}$ $cm^3\ s^{-1}$, which is in agreement with the second-order recombination rate constant of $(2.3 \pm 0.6) \times 10^{-9}$ $cm^3\ s^{-1}$ in organometallic halide perovskite films[12].

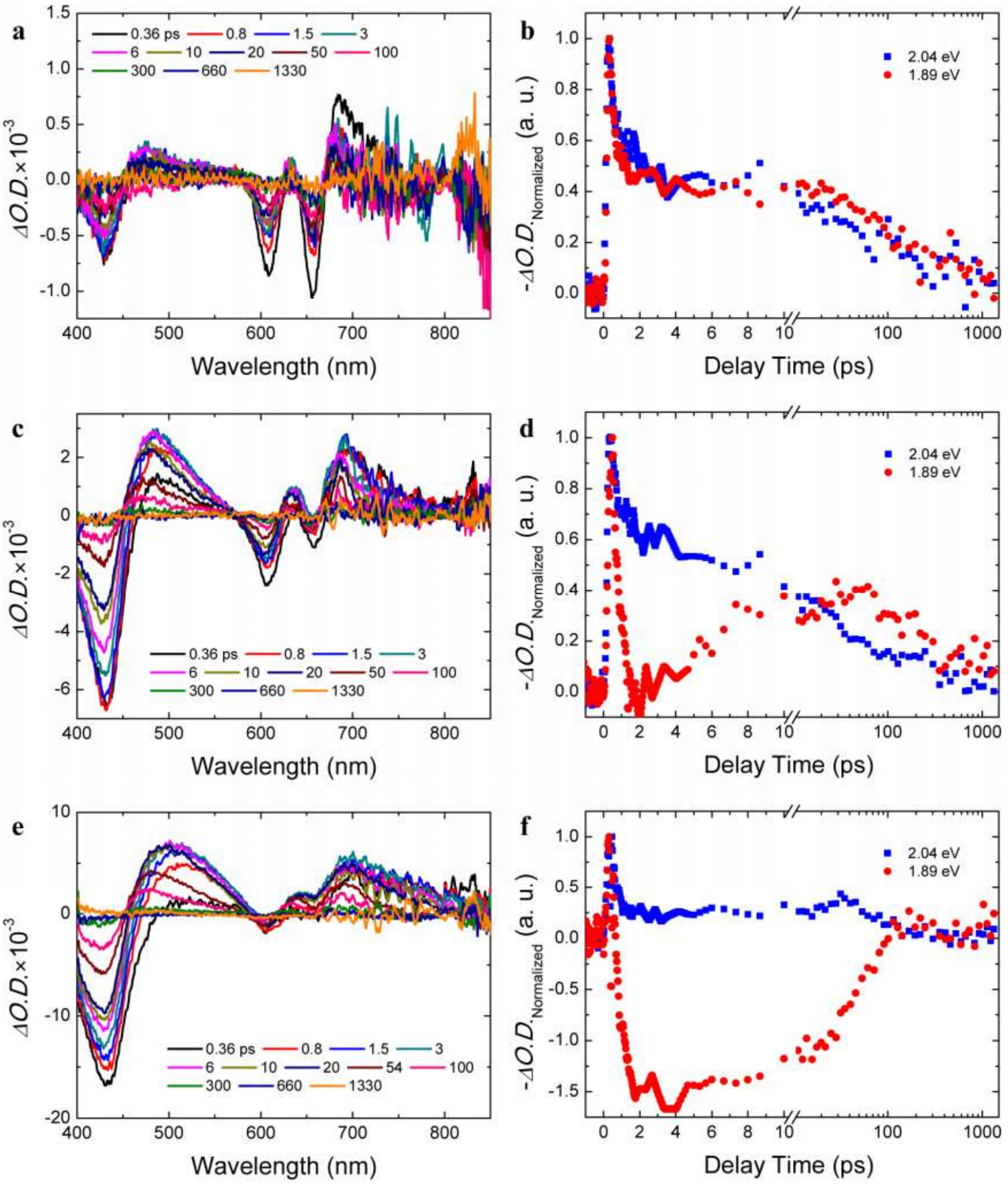

Supplementary Figure 12. TA spectra at a pump density of **a**, 5 μJ cm^{-2}, **c**, 80 μJ cm^{-2}, and **e**, 240 μJ cm^{-2} under 400 nm excitation. **b**, **d**, and **f** show, respectively, the

normalized dynamics of band-edge states at a pump density of 5 μJ cm^{-2}, 80 μJ cm^{-2}, and 240 μJ cm^{-2}.

Supplementary Note 5. At low pump density (case shown in Supplementary Figure 12a,b), all the transient signals are small (Supplementary Figure 12a), and an excited-state absorption signal in the red side of the band-edge state at 1.89 eV (656 nm) is observed. In this pump condition, the characterization dynamics of both band-edge states at 2.04 eV (608 nm) and 1.89 eV (656 nm) are similar (Supplementary Figure 12b), but when the pump density increases, the excited-state absorption signal becomes larger, and its influence on the band-edge states becomes more notorious (case shown in Supplementary Figure 12c,d). Further increasing the pump density (Supplementary Figure 12e,f), a broader excited-state absorption signal appears, covering the spectral range from the band-edge states to the near infrared region. Concerning the transient signal at the band-edge state at 1.89 eV (656 nm), it becomes positive as a result of the spectral overlapping with the stronger excited-state absorption and/or other signals from new photogenerated transient species, i.e., the charges[13,14] (Supplementary Figure 13).

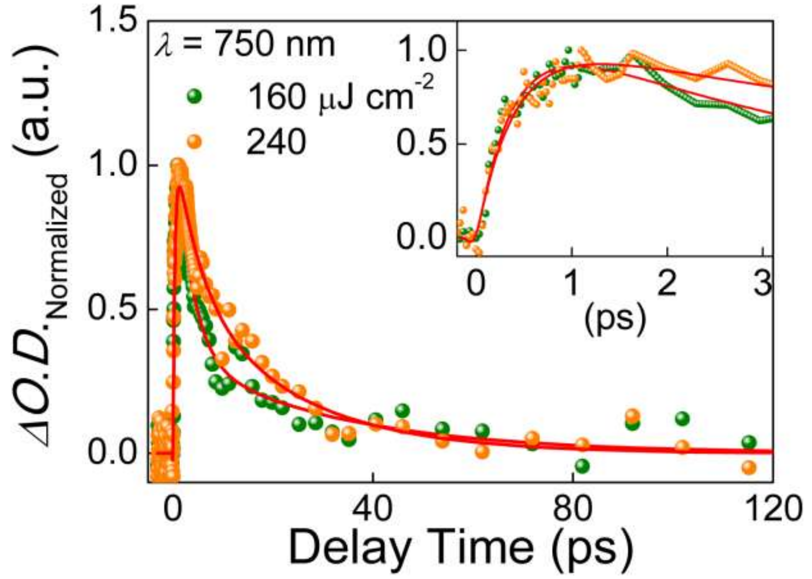

Supplementary Figure 13. Normalized decay dynamics of the excited-state absorption signal probed at 750 nm under 400 nm excitation at high pump densities. Inset is the dynamics curves within the first several picoseconds. Multi-exponential fitting gives a rise time of 0.37 ps (0.39 ps) and an average decay lifetime of 11 ps (14 ps) for pump density at 160 μJ cm^{-2} (240 μJ cm^{-2}). The two decay lifetime components are τ_1 = 3.3 ps (73%), τ_2 = 32 ps (27%) for pump density of 160 μJ cm^{-2}, and τ_1 = 5.2 ps (45%), τ_2 = 22 ps (55%) for pump density of 240 μJ cm^{-2}, respectively. Through the multi-exponential fitting, we deduce these processes could be assigned to the generation and decay of low-energy charges (compared with the hot carriers in the C-exciton state). As a matter of fact, the ~0.4 ps rise time obtained is consistent with the reported exciton dissociation time of ~0.7 ps in few-layer MoS_2 films[13].

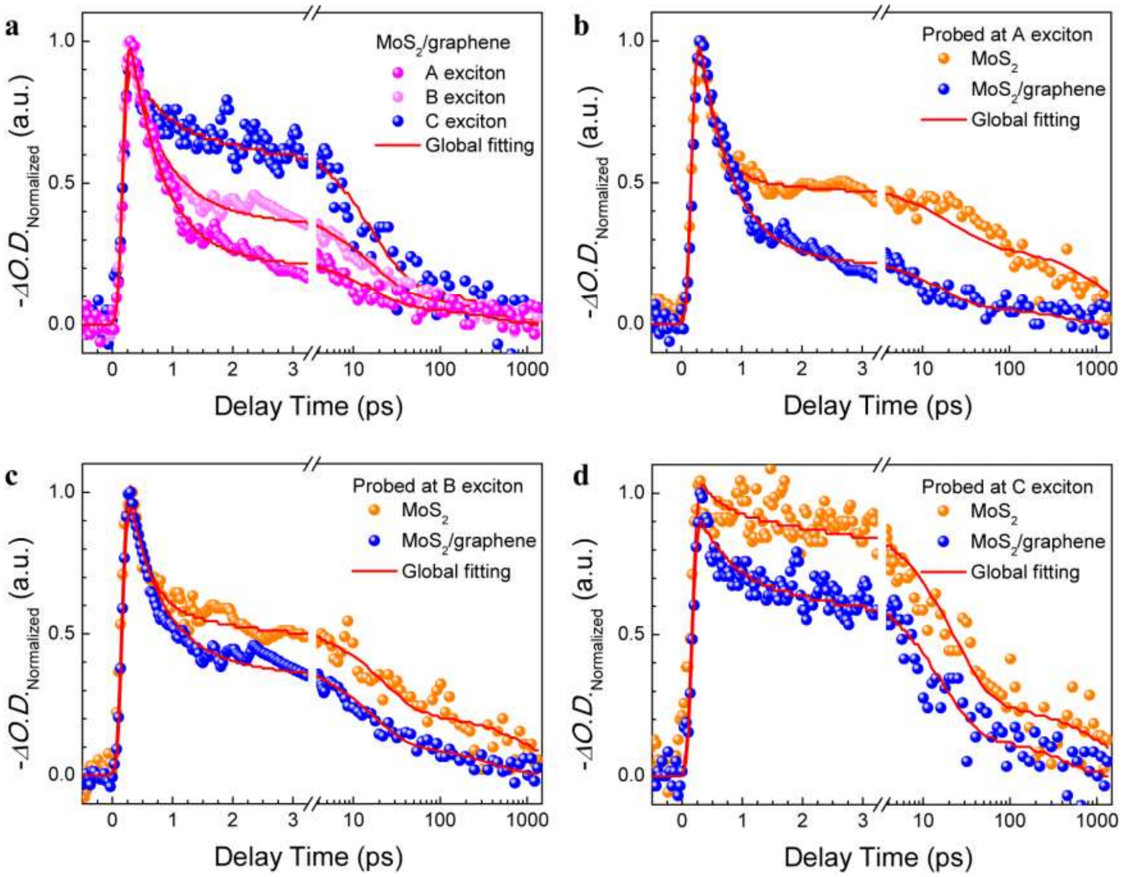

Supplementary Figure 14. a, Normalized decay dynamics of A-, B-, and C-exciton state for MoS_2 monolayer/graphene heterostructures under the pump condition in Fig. 4, panel **a**. A direct comparison for the exciton dynamics in the TA experiemts of MoS_2 with and without graphene substrate under 400 nm excitation. **b**, **c**, and **d** show, respectively, the normalized dynamics of A-, B- and C-exciton state. The red solid lines are the global fitting results.

Supplementary Table 1. Femtosecond time-resolved TA dynamics parameters obtained by global fitting under 400 nm excitation.

	Lifetime component	τ_1 (0.27 ps)	τ_2 (1.3 ps)	τ_3 (23 ps)	τ_4 (1500 ps)	τ_{ave} (ps)	
MoS_2 monolayer	A exciton	0.59	0.02	0.17	0.22	334 ± 10	
	B exciton	0.54	0.06	0.24	0.16	246 ± 8	
	C exciton	0.14	0.04	0.59	0.23	360 ± 12	
	Lifetime component		τ_1 (0.55 ps)	τ_2 (16 ps)	τ_3 (420 ps)	τ_{ave} (ps)	η
MoS_2 monolayer /Graphene	A exciton		0.80	0.15	0.05	24 ± 1	0.93 ± 0.01
	B exciton		0.63	0.27	0.10	47 ± 1	0.81 ± 0.01
	C exciton		0.30	0.55	0.15	72 ± 2	0.80 ± 0.01

carrier extraction efficiency: $\eta = 1 - \tau_{MoS_2-Graphene} / \tau_{MoS_2}$

Supplementary References

1. Lee, C. G. *et al*. Anomalous lattice vibrations of single- and few-layer MoS_2. *ACS Nano* **4**, 2695–2700 (2010).
2. Ma, Y. D. *et al*. Graphene adhesion on MoS_2 monolayer: An *ab initio* study. *Nanoscale*, **3**, 3883–3887 (2011).
3. Ye, X. H. *et al*. Lap joining of graphene flakes by current-assisted CO_2 laser irradiation. *Carbon* **61**, 329–335 (2013).
4. Carozo, V. *et al*. Resonance effects on the Raman spectra of graphene superlattices. *Phys. Rev. B* **88**, 085401 (2013).
5. Jorio, A. *et al*. Optical-phonon resonances with saddle-point excitons in twisted-bilayer graphene. *Nano Lett.* **14**, 5687–5692 (2014).
6. Havener, R. W. *et al*. Hyperspectral imaging of structure and composition in atomically thin heterostructures. *Nano Lett.* **13**, 3942–3946 (2013).
7. Kumar, N. *et al*. Charge carrier dynamics in bulk MoS_2 crystal studied by transient absorption microscopy. *J. Appl. Phys.* **113**, 133702 (2013).
8. Qiu, D. Y. *et al*. Optical spectrum of MoS_2: many-body effects and diversity of exciton states. *Phys. Rev. Lett.* **111**, 216805 (2013).
9. Sun, D. Z. *et al*. Observation of rapid exciton-exciton annihilation in monolayer molybdenum disulfide. *Nano Lett*. **15**, 2992–2997 (2015).

10. Chernikov, A. *et al*. Exciton binding energy and nonhydrogenic Rydberg series in monolayer WS_2. *Phys. Rev. Lett.* **113**, 076802 (2014).

11. He, K. L. *et al*. Tightly bound excitons in monolayer WSe_2. *Phys. Rev. Lett.* **113**, 026803 (2014).

12. Manser, J. S. & Kamat, P. V. Band filling with free charge carriers in organometal halide perovskites. *Nat. Photon.* **8**, 737−743 (2014).

13. Borzda, T. *et al*. Charge photogeneration in few-layer MoS_2. *Adv. Funct. Mater.* **25**, 3351−3358 (2015).

14. Chernikov, A. *et al*. Population inversion and giant bandgap renormalization in atomically thin WS_2 layers. *Nature Photon.* **9**, 466−470 (2015).
